# Supplementary material for: Cell-type-specific genomics reveals histone modification dynamics in mammalian meiosis
Source: Nat Commun. 2019 Aug 23;10:3821. doi: 10.1038/s41467-019-11820-7 (PMC6707301; doi:10.1038/s41467-019-11820-7)
Supplement: Supplementary file 1 — Supplementary Information [file 41467_2019_11820_MOESM1_ESM.pdf]

Cell-type-specific genomics reveals histone modification dynamics in mammalian meiosis

Lam et al.

Supplementary Figures

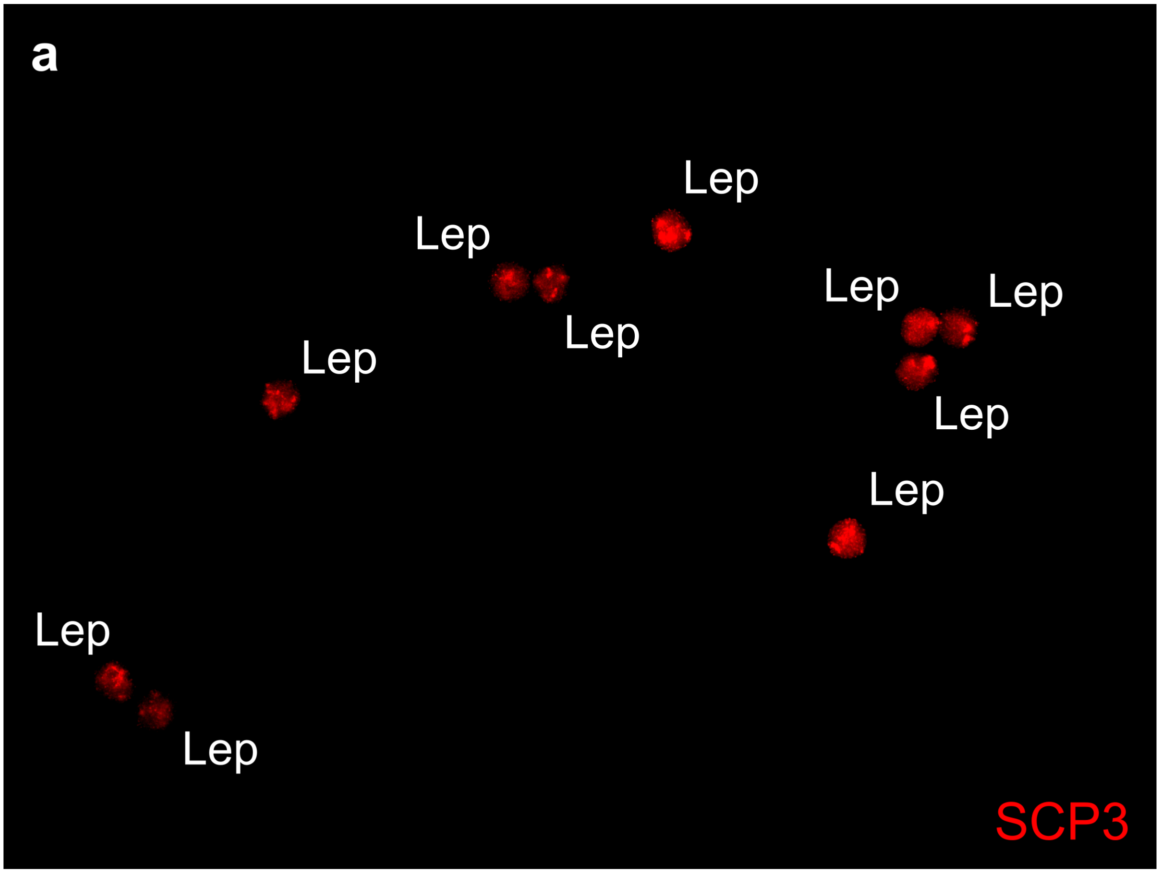

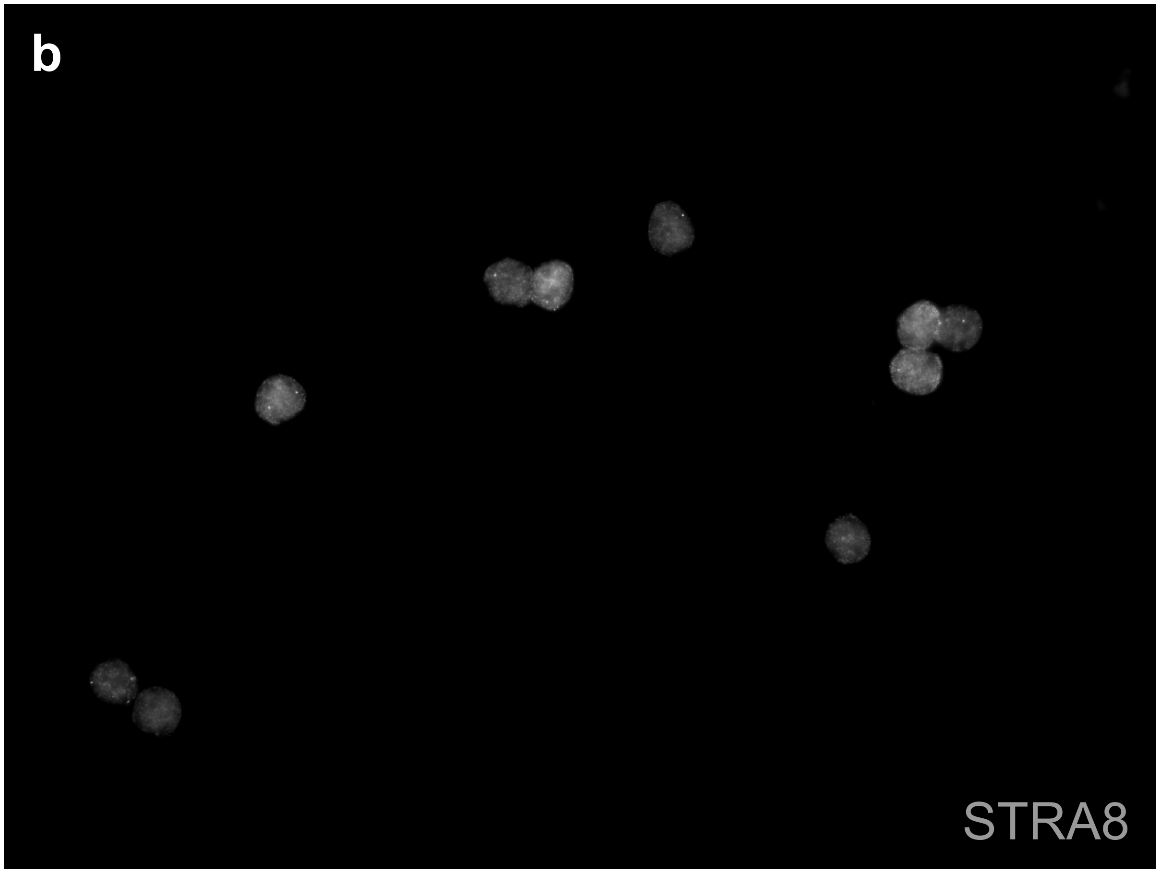
b
STRA8

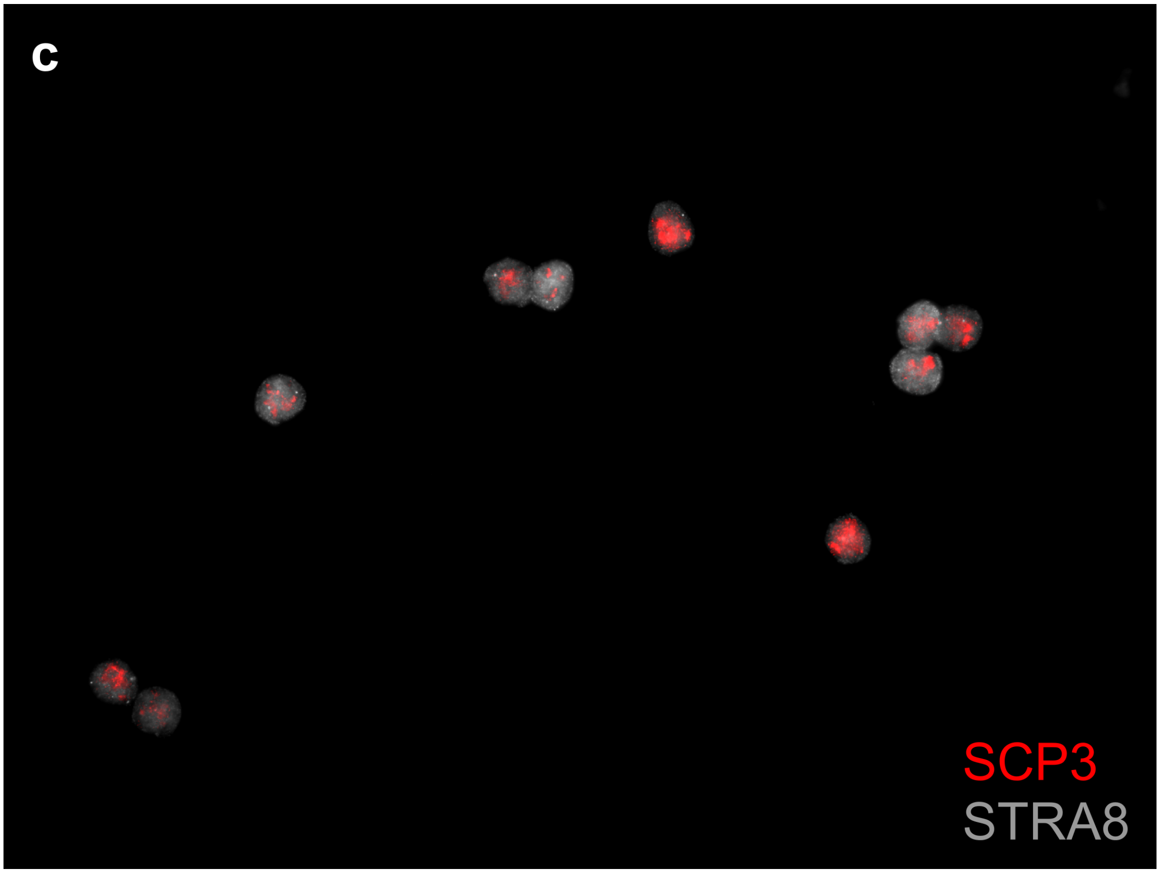
c
SCP3
STRA8

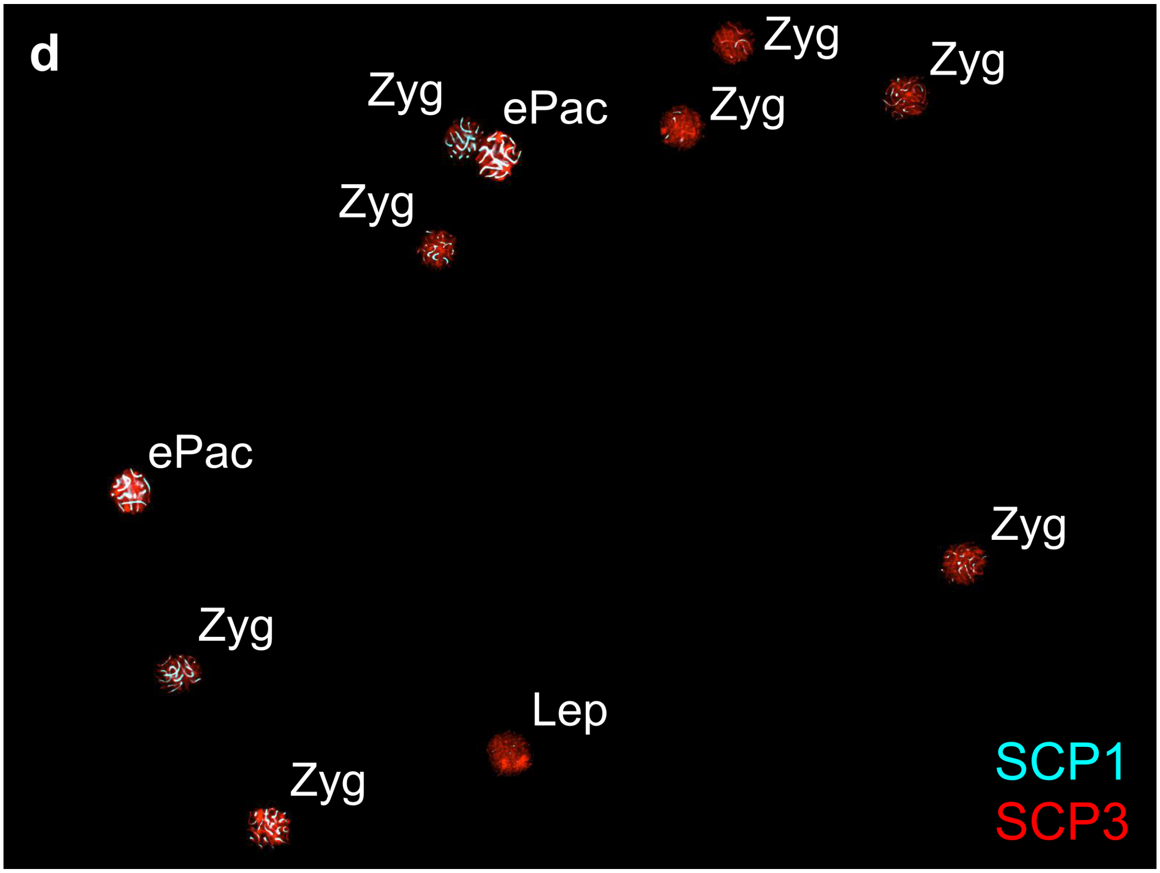
d
Zyg
Zyg
ePac
Zyg
Zyg
Zyg
ePac
Zyg
Zyg
Lep
Zyg
SCP1
SCP3

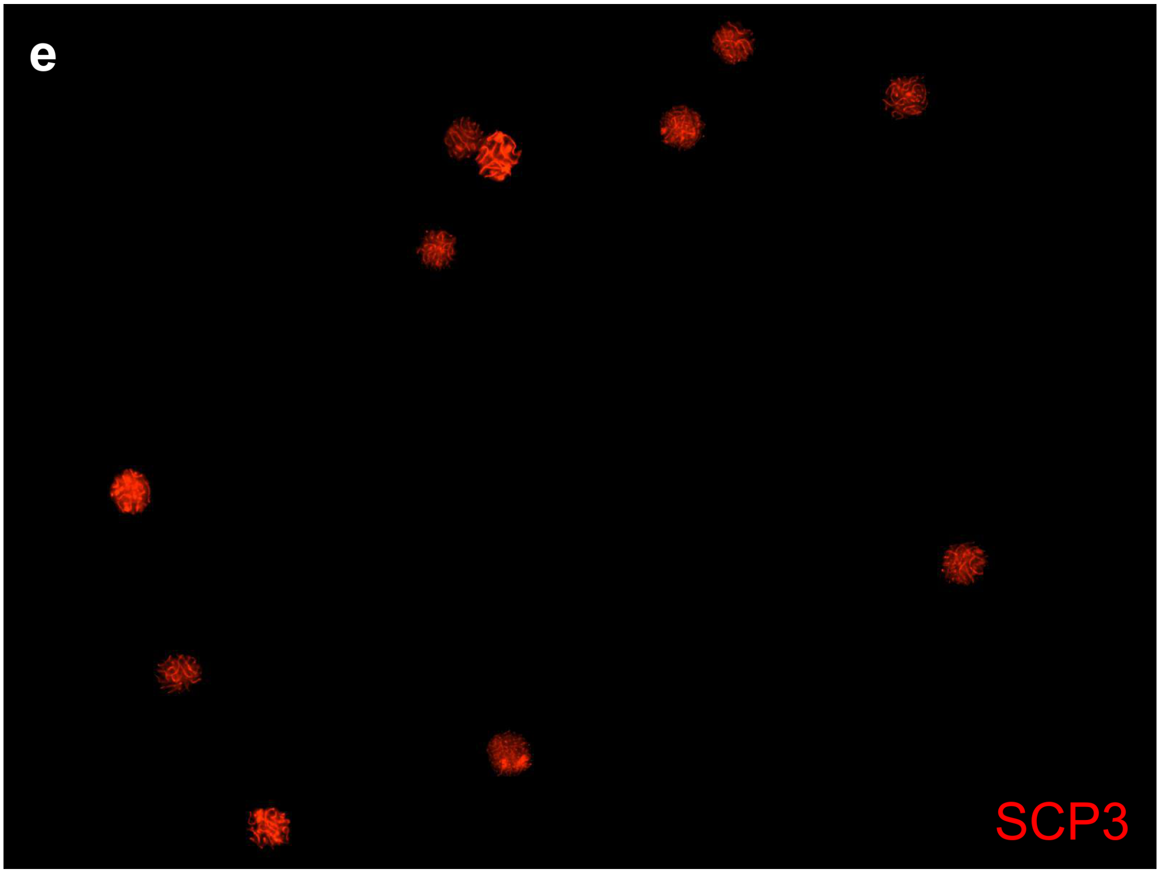
e
SCP3

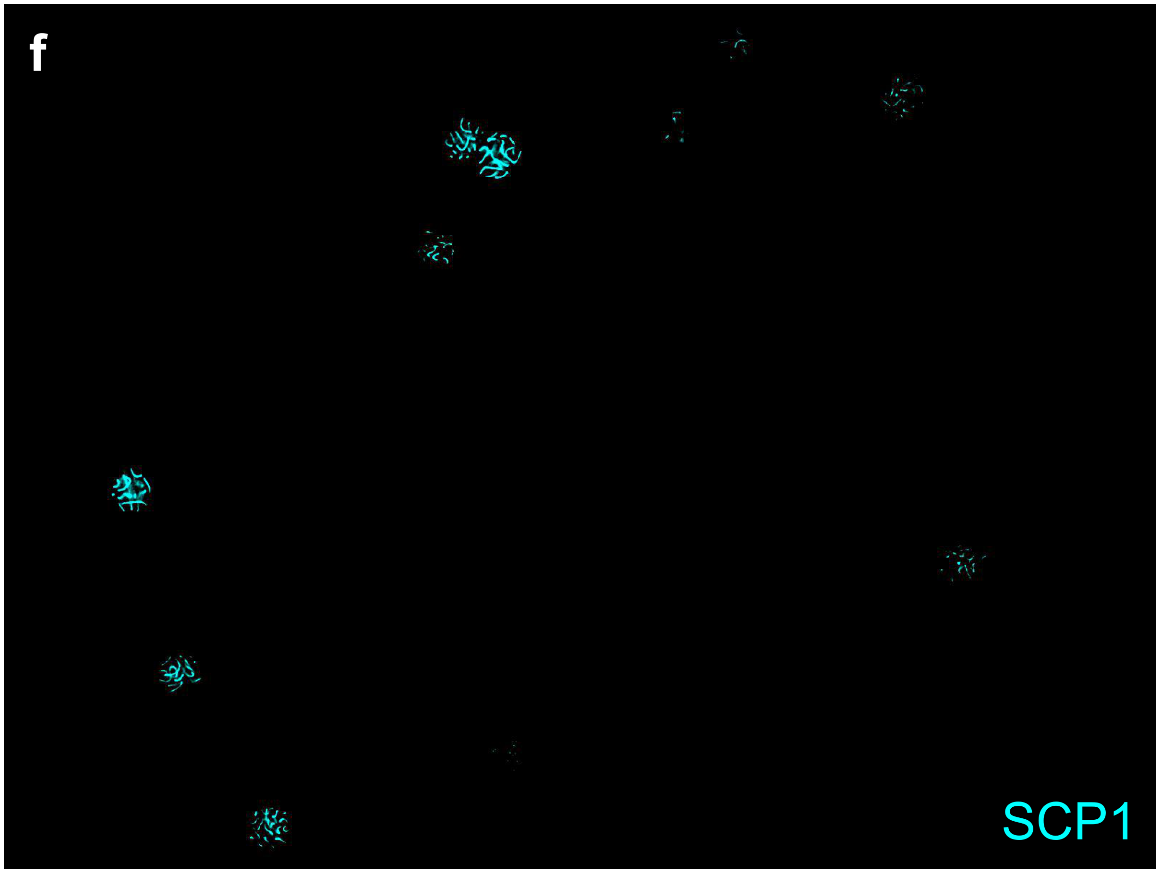

Supplementary Fig. 1 Microscopic examination of sorted nuclei after FACS. Whole-field immunofluorescence images of sorted nuclei in the populations of **a**-**c** leptonema and **d**-**f** zygonema. Lep=leptonema; Zyg=zygonema; ePac=early pachynema.

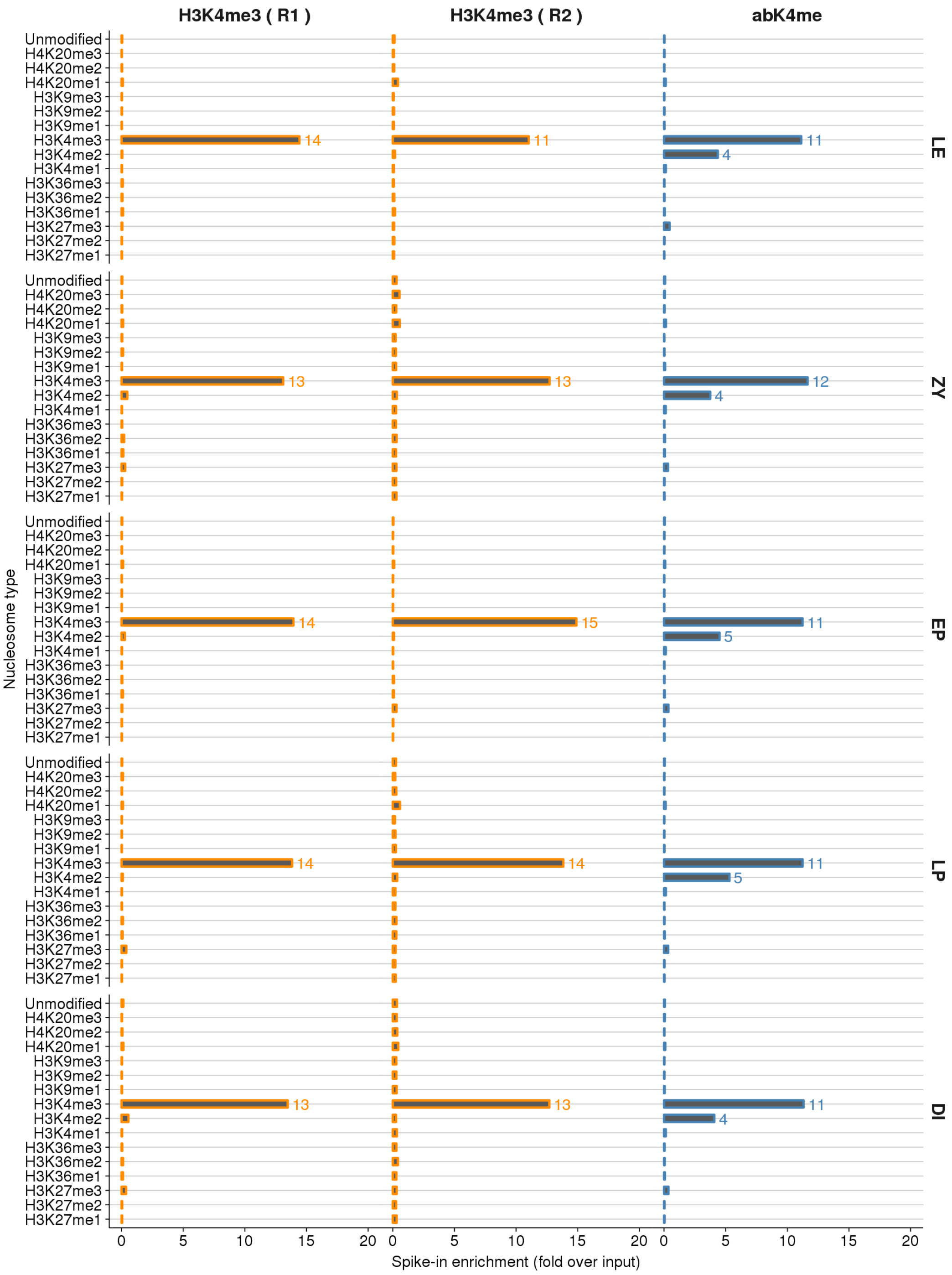

Supplementary Fig. 2 H3K4me3 antibodies have varying levels of substrate specificity.

We performed ChIP-Seq across five MPI stages using two H3K4me3 antibodies; EpiCypher 13-0028 (H3K4me3; two replicates) and Abcam ab8580 (abK4me). The EpiCypher K-MetStat panel is a collection of nucleosomes with different histone modifications that was spiked into each H3K4me3 ChIP-Seq experiment

to validate the specificity of the antibody and to allow cross sample normalization for ChIP-efficiency (see methods). The relative enrichment of sequences bound to each modified nucleosome are shown here, compared to the relative frequency in the sample before ChIP was performed. The Epicypher antibody is highly specific to H3K4me3, however, as previously demonstrated[1], the Abcam antibody shows substantial cross-reactivity with H3K4me2. Note that although the enrichment for H3K4me2 is relatively low compared to H3K4me3, the relative abundance of H3K4me2 in the genome compared to H3K4me3[1] makes it difficult to assess the quantitative impact of this cross-reactivity in ChIP-Seq experiments.

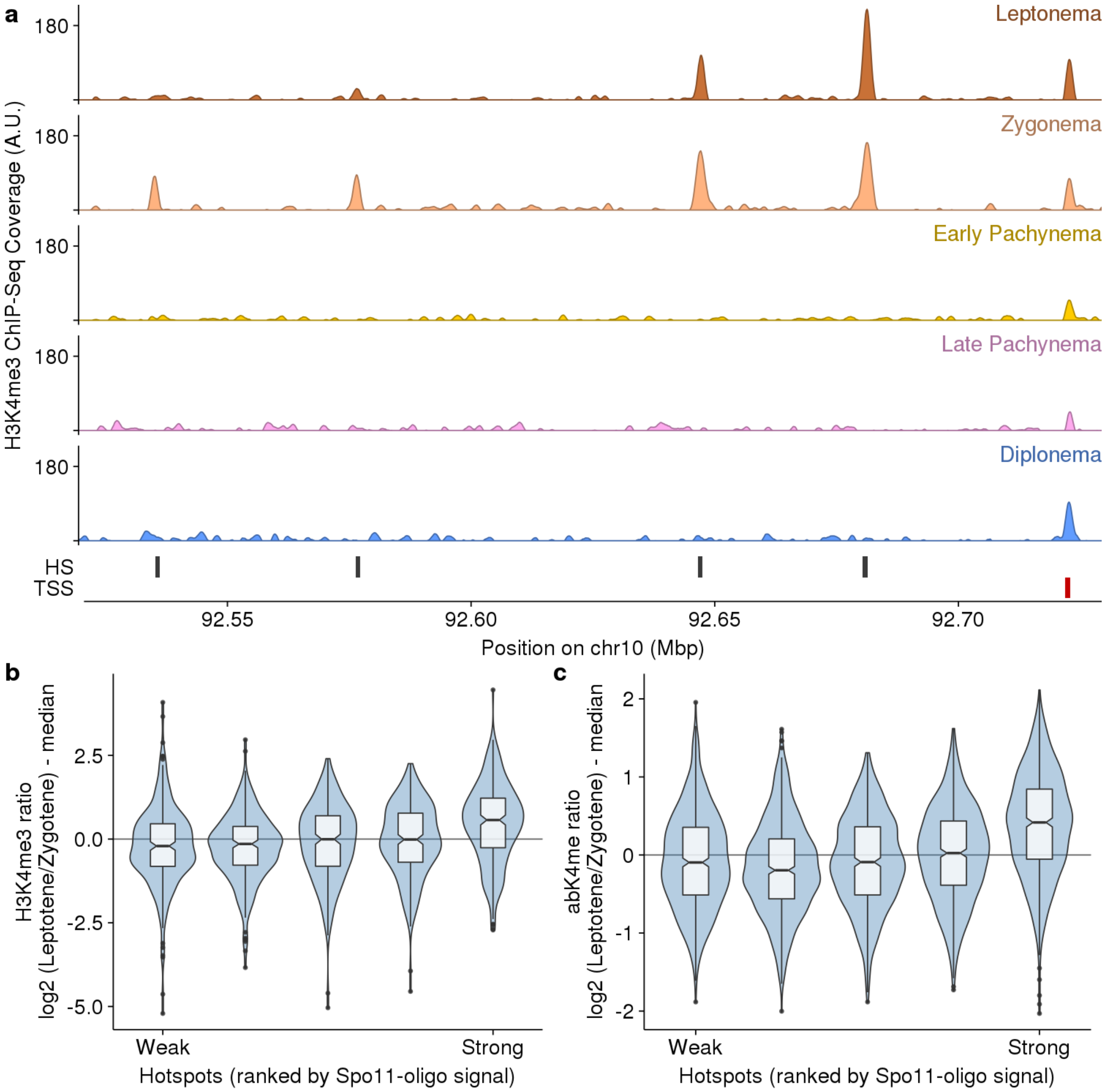

Supplementary Fig. 3 H3K4me3 is relatively elevated at stronger DSB hotspots in leptonema. **a** Snapshot of H3K4me3 at four DSB hotspots. The DSB hotspots are progressively stronger from left to right. At the three weaker hotspots (left) the H3K4me3 signal is maximal at zygonema. At the strongest hotspot (right) the H3K4me3 signal is strongest at leptonema. **b-c** Systematic trend towards stronger leptonema H3K4me3 at stronger DSB hotspots. **b** The H3K4me3 ratio between leptonema and zygonema is shown. **c** A more pronounced trend is seen using the Abcam ab8580 H3K4me3 antibody (abK4me).

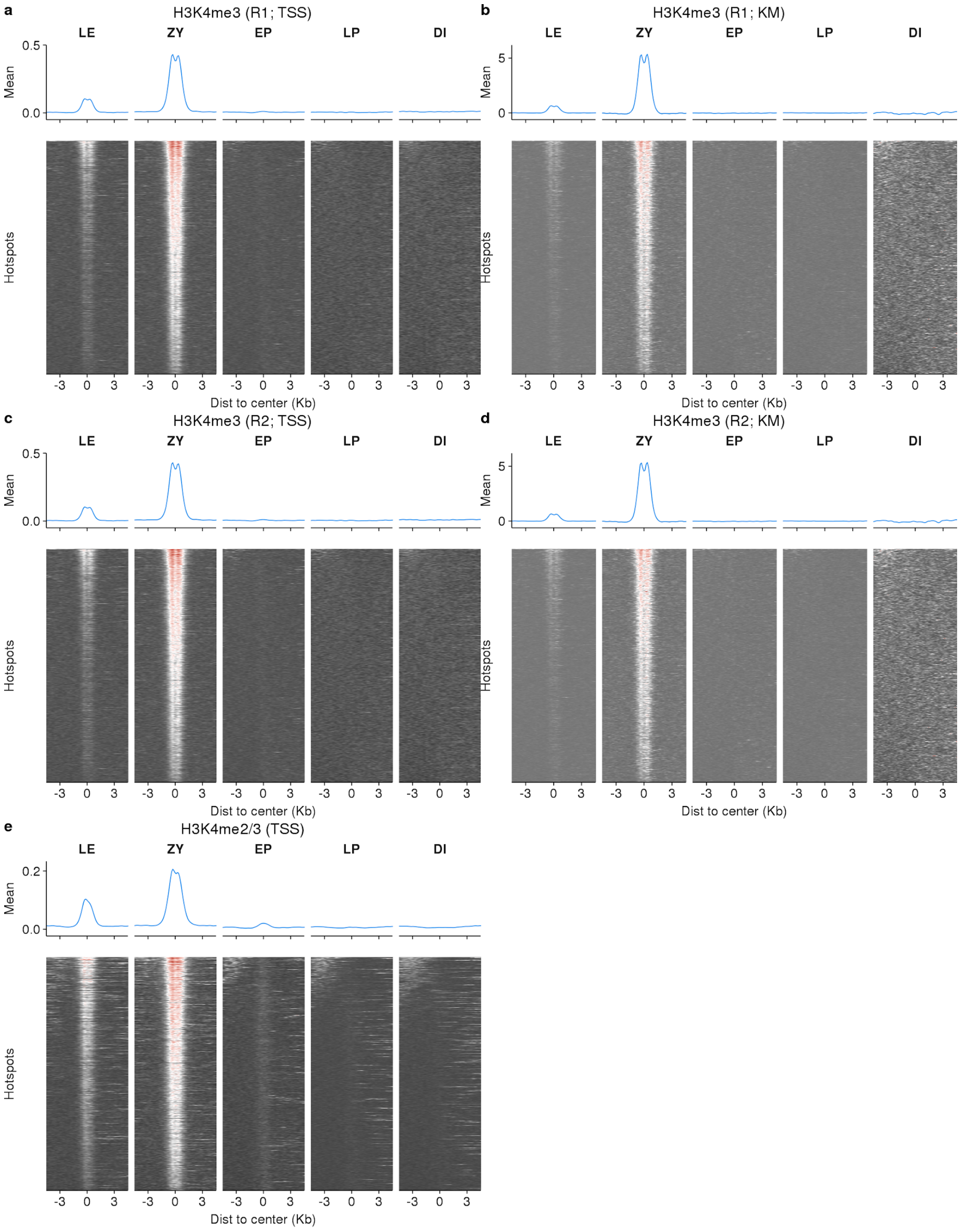

Supplementary Fig. 4 H3K4me3 dynamics are similar using different normalization approaches.

Coverage heatmaps at DSB hotspots through MPI. **a,c,e** Coverage is normalized using NCIS and stable TSS signals (see methods). **b,d** Coverage is normalized

using the K-MetStat spike-in. The spike-in normalization cannot be used for the H3K4me2/3 sample because of antibody cross-reactivity (Abcam ab8580 antibody). Both normalization methods yield similar results for H3K4me3 replicate 1 (R1; **a,b**) and replicate 2 (R2; **c,d**). A slight increase in the leptonema signal and a marginal signal at early pachynema are seen with the H3K4me2/3 ChIP-Seq (**e**).

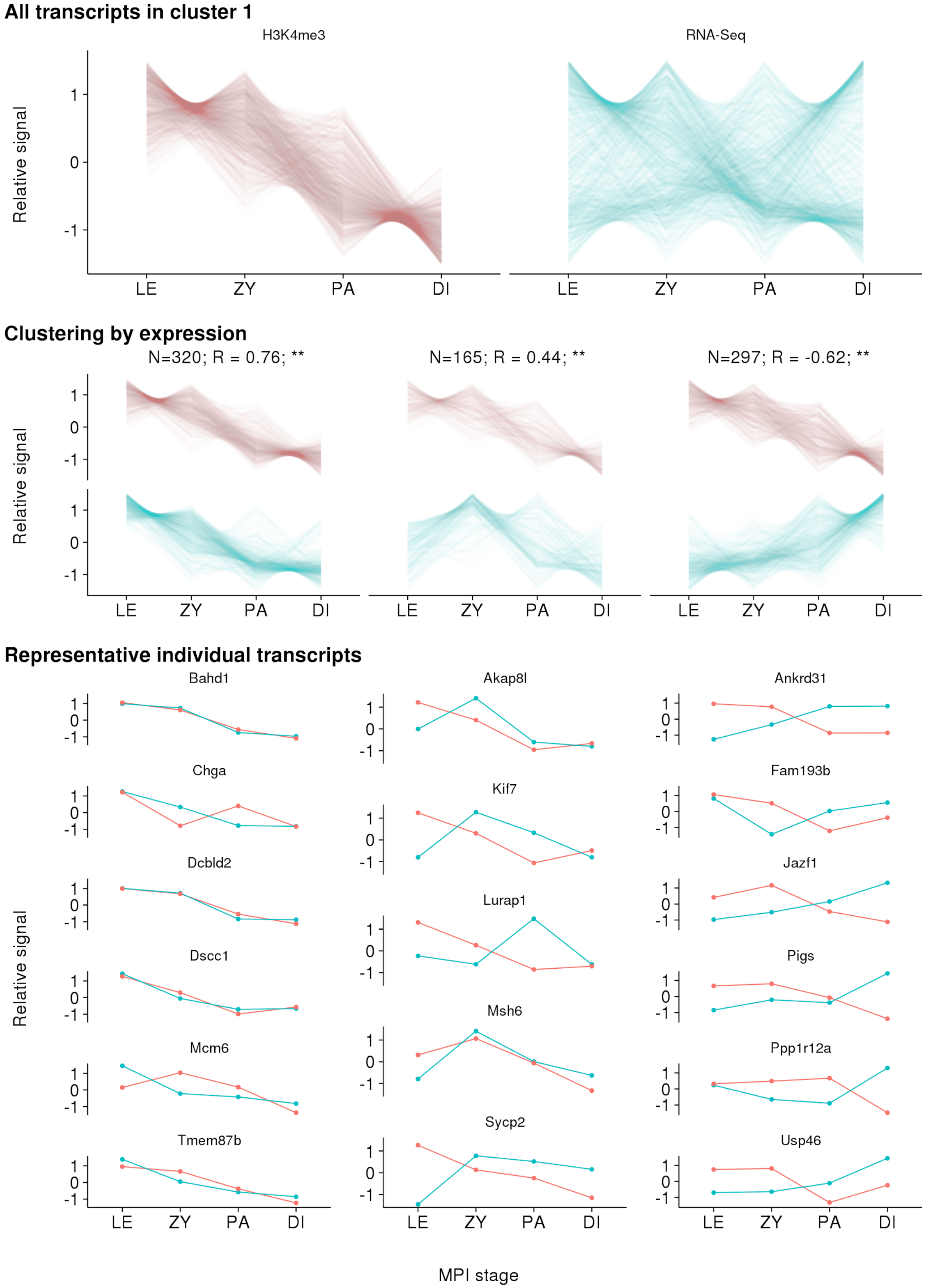
All transcripts in cluster 1
H3K4me3
RNA-Seq
Relative signal
1
0
-1
LE
ZY
PA
DI
Clustering by expression
N=320; R = 0.76; **
N=165; R = 0.44; **
N=297; R = -0.62; **
Representative individual transcripts
Bahd1
Akap8l
Ankrd31
Chga
Kif7
Fam193b
Dcbld2
Jazf1
Lurap1
Dscc1
Pigs
Msh6
Mcm6
Ppp1r12a
Tmem87b
Sycp2
Usp46
MPI stage

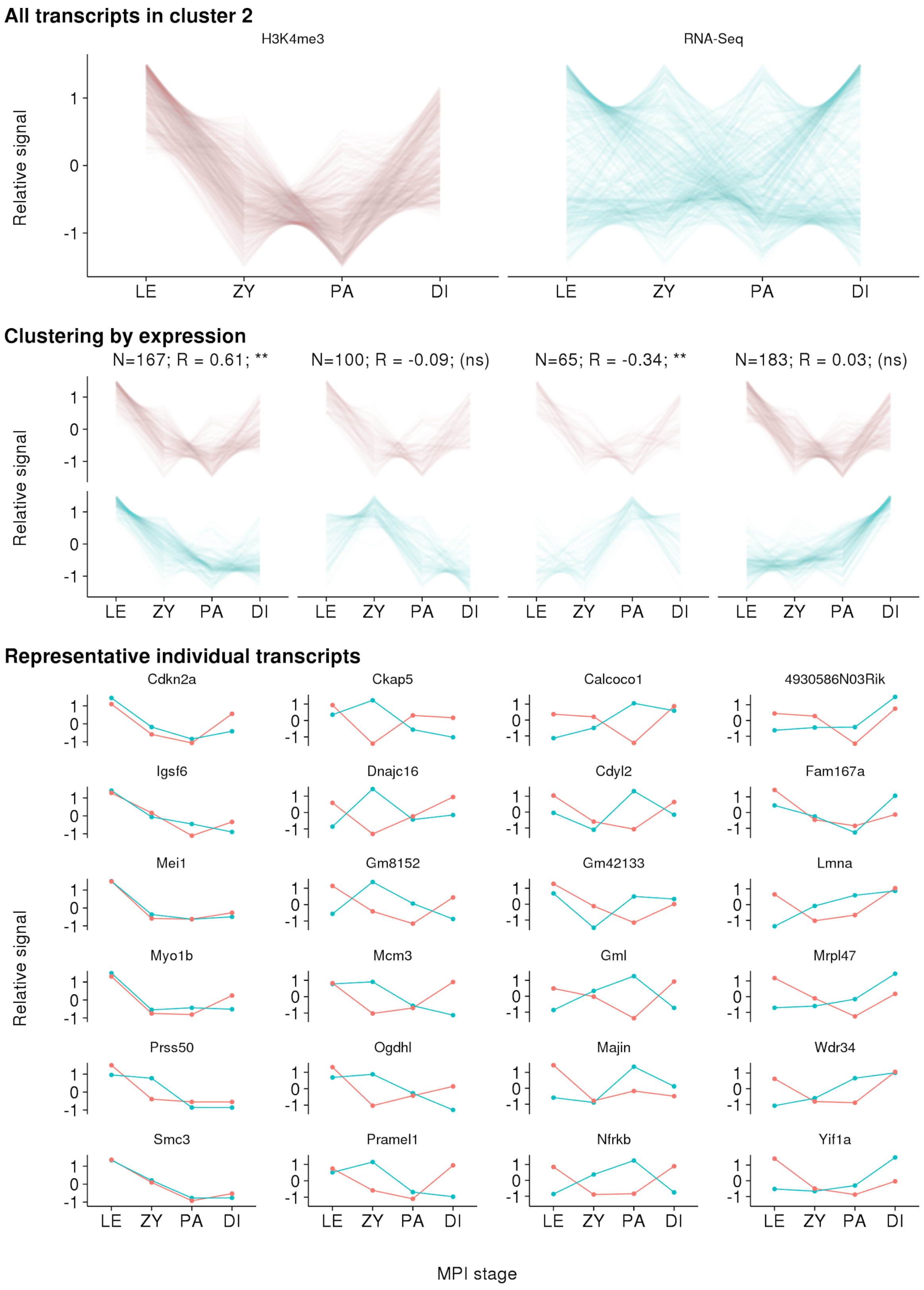
All transcripts in cluster 2
H3K4me3
RNA-Seq
Relative signal
LE
ZY
PA
DI
Clustering by expression
N=167; R = 0.61; **
N=100; R = -0.09; (ns)
N=65; R = -0.34; **
N=183; R = 0.03; (ns)
Representative individual transcripts
Cdkn2a
Ckap5
Calcoco1
4930586N03Rik
Igsf6
Dnajc16
Cdyl2
Fam167a
Mei1
Gm8152
Gm42133
Lmna
Myo1b
Mcm3
Gml
Mrpl47
Prss50
Ogdhl
Majin
Wdr34
Smc3
Pramel1
Nfrkb
Yif1a
MPI stage

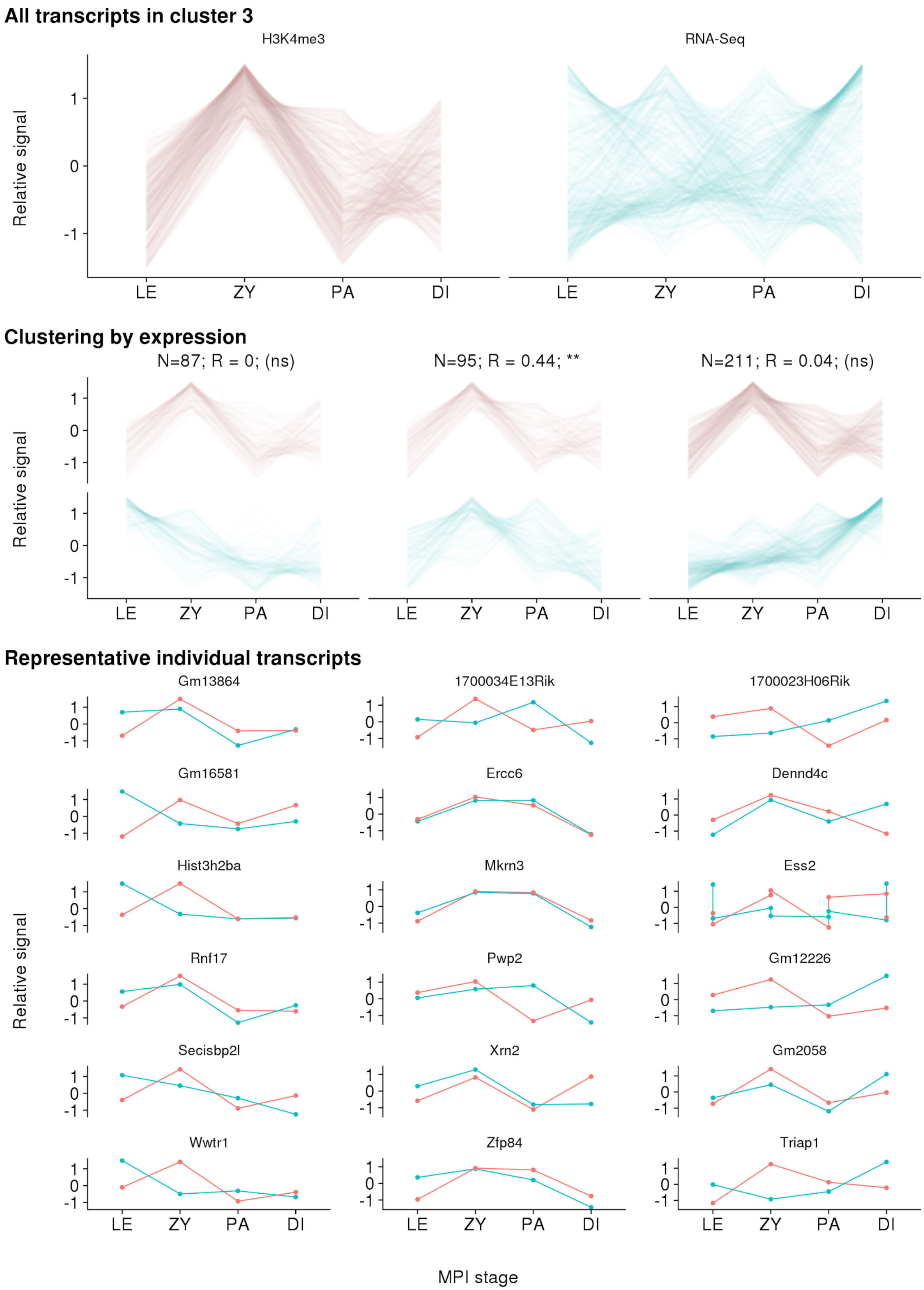
All transcripts in cluster 3
H3K4me3
RNA-Seq
Relative signal
LE
ZY
PA
DI
Clustering by expression
N=87; R = 0; (ns)
N=95; R = 0.44; **
N=211; R = 0.04; (ns)
Representative individual transcripts
Gm13864
1700034E13Rik
1700023H06Rik
Gm16581
Ercc6
Dennd4c
Hist3h2ba
Mkrn3
Ess2
Rnf17
Pwp2
Gm12226
Secisbp2l
Xrn2
Gm2058
Wwtr1
Zfp84
Triap1
MPI stage

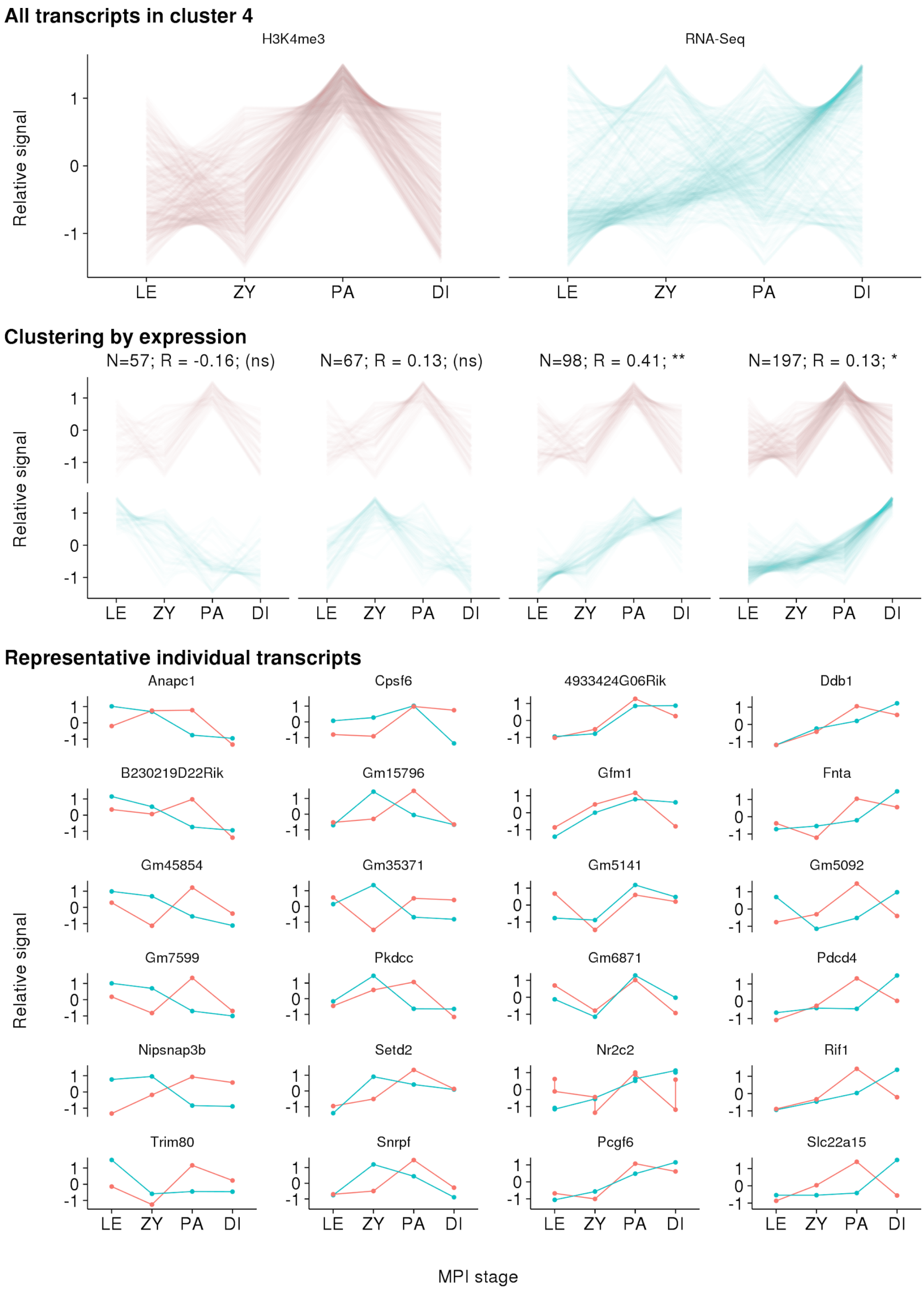

All transcripts in cluster 4
H3K4me3
RNA-Seq
Relative signal
LE
ZY
PA
DI
Clustering by expression
N=57; R = -0.16; (ns)
N=67; R = 0.13; (ns)
N=98; R = 0.41; **
N=197; R = 0.13; *
Representative individual transcripts
Anapc1
Cpsf6
4933424G06Rik
Ddb1
B230219D22Rik
Gm15796
Gfm1
Fnta
Gm45854
Gm35371
Gm5141
Gm5092
Gm7599
Pkdcc
Gm6871
Pdcd4
Nipsnap3b
Setd2
Nr2c2
Rif1
Trim80
Snrpf
Pcgf6
Slc22a15
MPI stage

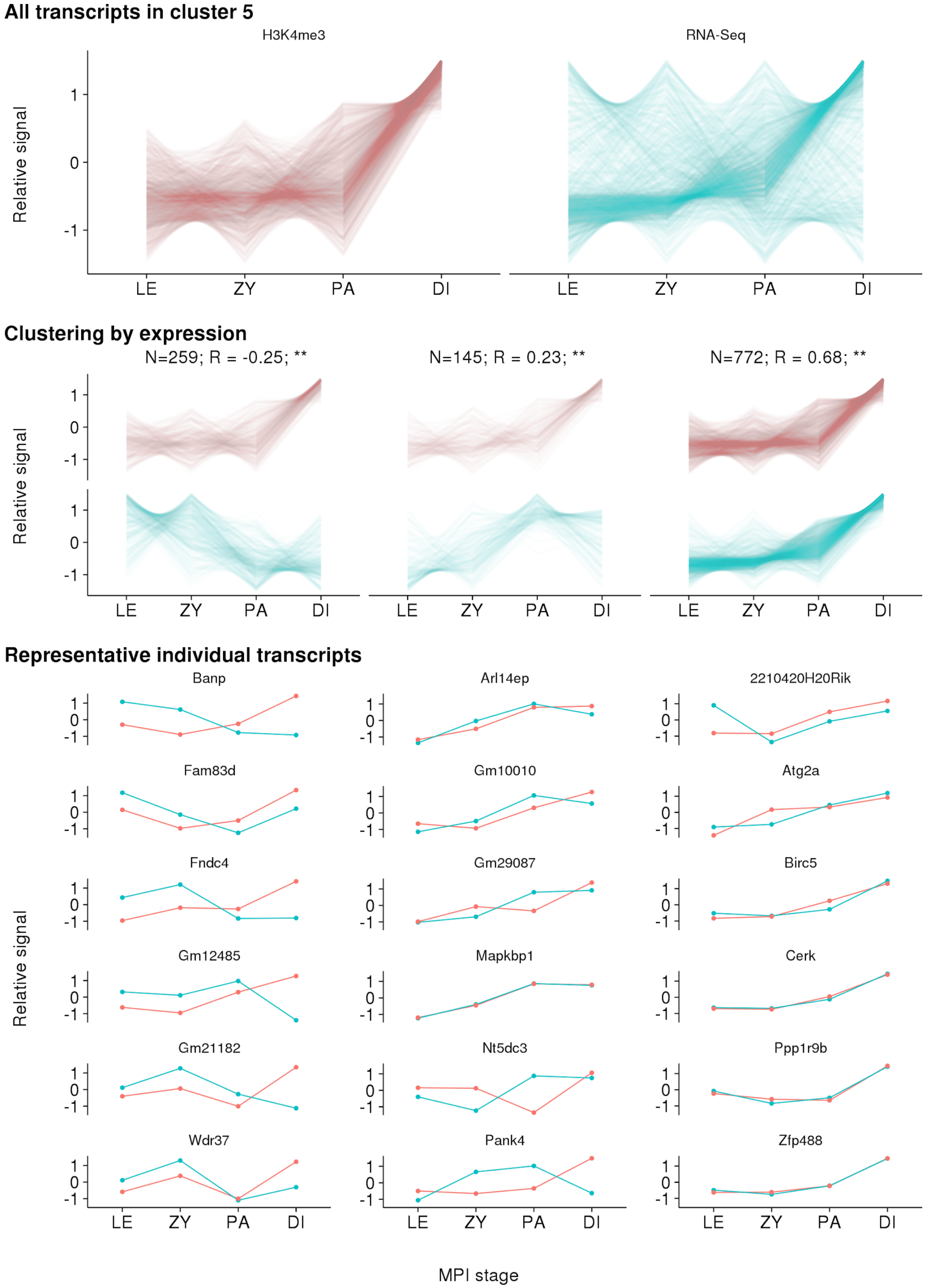

All transcripts in cluster 5
H3K4me3
RNA-Seq
Relative signal
LE
ZY
PA
DI
Clustering by expression
N=259; R = -0.25; **
N=145; R = 0.23; **
N=772; R = 0.68; **
Representative individual transcripts
Banp
Arl14ep
2210420H20Rik
Fam83d
Gm10010
Atg2a
Fndc4
Gm29087
Birc5
Gm12485
Mapkbp1
Cerk
Gm21182
Nt5dc3
Ppp1r9b
Wdr37
Pank4
Zfp488
MPI stage

Supplementary Fig. 5 H3K4me3 dynamics correlate with gene expression profiles from male juvenile mice.

Each of the plots represents a single cluster of transcripts from Fig. 3 (Cluster 1-5 are from left to right on Fig. 3c, respectively). Throughout, red depicts H3K4me3 ChIP-Seq signal and blue depicts gene expression from RNA-Seq. Uppermost panels show all transcripts in the cluster; lines represent individual transcripts. Transcripts were next sub-clustered by gene expression profile (middle panels; k-means clustering; optimal k chosen using gap statistic). Clusters in which profiles correlate more or less than expected are highlighted (* = $P < 0.01$, ** = $P < 0.001$; 1,000 bootstraps of shuffling stages for each gene). Six representative transcripts from each cluster are shown in the lower panel below each cluster. Transcripts of genes known to play a role in MPI were preferentially selected; if < 6 such genes were present in a cluster, the remainder were randomly selected.

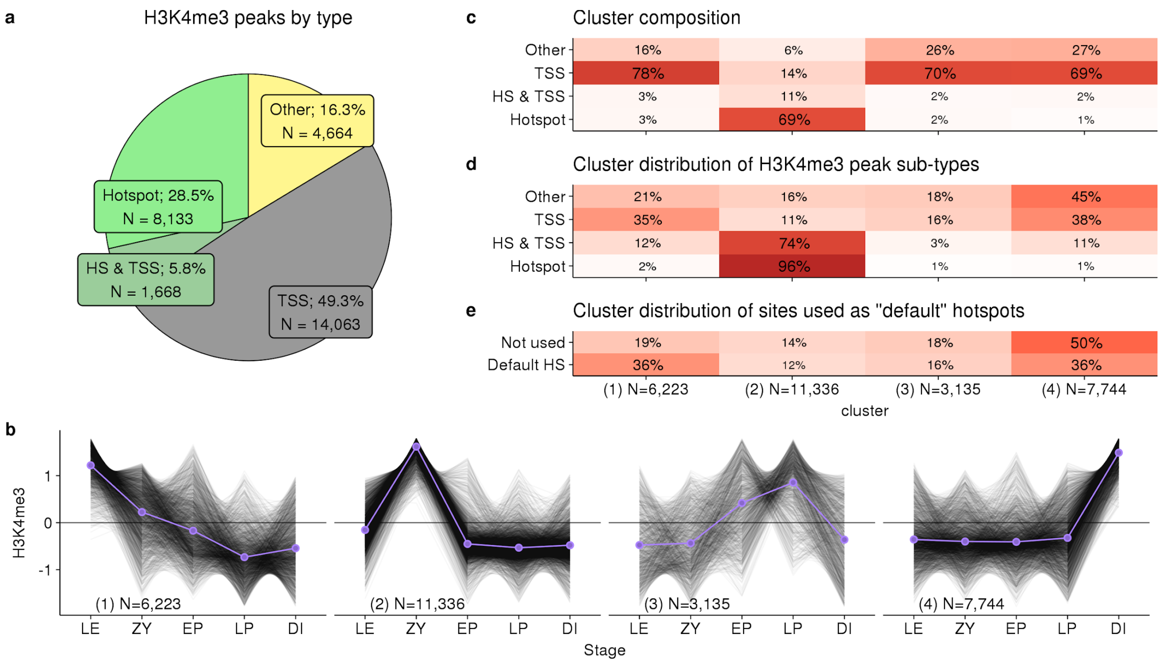

Supplementary Fig. 6 Extensive dynamics revealed by unbiased clustering of H3K4me3 peaks. a 16% of all H3K4me3 peaks occur at sites not defined at DSB hotspots or transcription start sites (TSS). These are classified as “Other”. 5.8% of H3K4me3 peaks coincide with both a DSB hotspot and a GENCODE TSS (HS & TSS). **b** Unbiased k-means clustering of MPI H3K4me3 profiles. Cluster number is indicated in parentheses in the bottom left corner. Each black line represents the MPI profile of an individual H3K4me3 peak. 10,000 randomly chosen profiles are shown. Purple lines depict the mean profile for each cluster. Profiles are normalized by the mean and standard deviation. The optimum number of clusters was identified using the gap statistic (optimum k = 5; range tested = 2 < k < 24). Cluster number is shown on each panel in parentheses. **c** Cluster composition shown as the percentage of each type of H3K4me3 peak within each cluster. Cluster 2 (N=11,336) is the only cluster composed primarily of peaks at DSB hotspots. **d** The percentage of peaks of each type across clusters is shown. Notably, 96% of DSB hotspots occur in cluster 2. 74% of HS & TSS peaks also occur in this cluster, suggesting that H3K4me3 at many of these sites is PRDM9-mediated. **e** In mice lacking PRDM9, PRDM9-independent H3K4me3 peaks are used for targeting the DSB machinery. These sites are termed “default hotspots” and are defined as H3K4me3 peaks that coincide with a DSB hotspot in *Prdm9*$^{-/-}$ mice[2]. Default hotspots form more frequently than expected at H3K4me3 peaks that are present in early MPI (cluster 1; N = 6,223). Peaks with a late dynamic (cluster 4; N=7,744) are used less than expected. The “Not used” peaks are all H3K4me3 peaks that do not coincide with either a PRDM9-defined or a default hotspot.

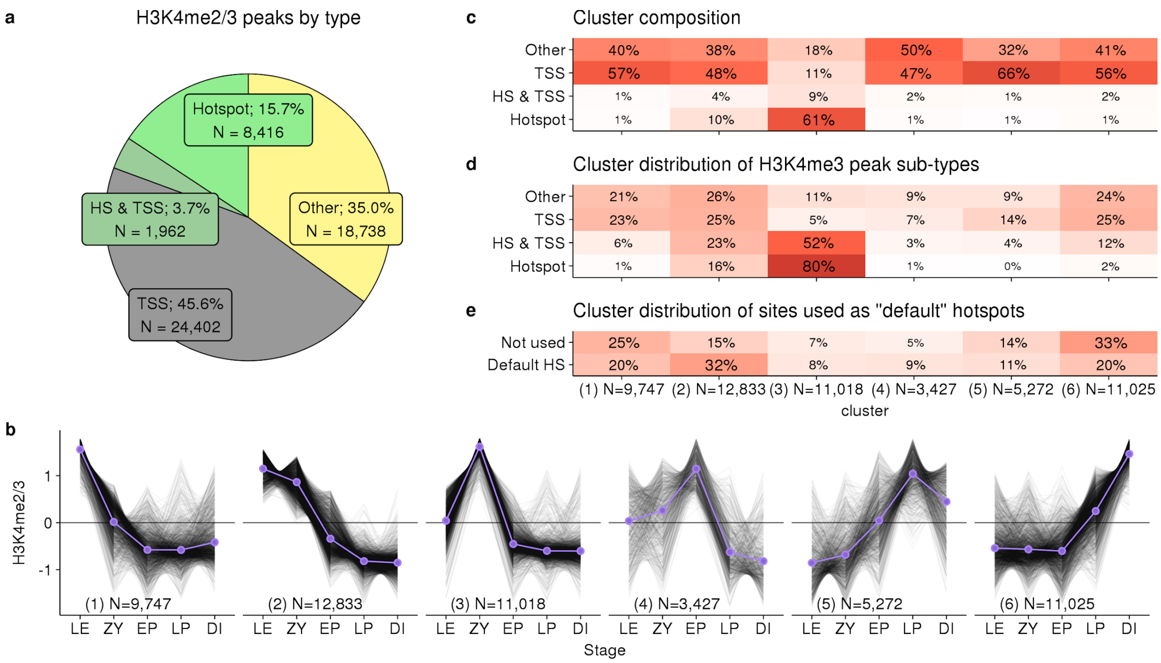

Supplementary Fig. 7 Extensive dynamics revealed by unbiased clustering of H3K4me2/3 peaks. a 35% of all H3K4me2/3 peaks (using Abcam ab8580 antibody) occur at sites not defined at DSB hotspots or transcription start sites (TSS). These are classified as “Other”. 3.7% of H3K4me3 peaks coincide with both a DSB hotspot and a GENCODE TSS (HS & TSS). **b** Unbiased k-means clustering of MPI H3K4me2/3 profiles. Cluster number is indicated in parentheses in the bottom left corner. Each black line represents the MPI profile of an individual H3K4me2/3 peak. 10,000 randomly chosen profiles are shown. Purple lines depict the mean profile for each cluster. Profiles are normalized by the mean and standard deviation. The optimum number of clusters was identified using the gap statistic (optimum k = 6; range tested = 2 < k < 24). Cluster number is shown on each panel in parentheses. **c** Cluster composition shown as the percentage of each type of H3K4me2/3 peak within each cluster. Cluster 3 is the only cluster composed primarily of peaks at DSB hotspots. **d** The percentage of peaks of each type across clusters is shown. Notably, 80% of DSB hotspots occur in cluster 3. 52% of HS & TSS peaks also occur in this cluster, suggesting that H3K4me3 at many of these sites is PRDM9-mediated. **e** In mice lacking PRDM9, PRDM9-independent H3K4me3 peaks are used for targeting the DSB machinery. These sites are termed “default hotspots” and are defined as H3K4me3 peaks that coincide with a DSB hotspot in *Prdm9*$^{-/-}$ mice[2]. Default hotspots form more frequently than expected at H3K4me3 peaks that are present in early MPI (cluster 2). Peaks with a late dynamic (cluster 5, 6) are used less than expected. “Not used” peaks are all H3K4me3 peaks that do not coincide with either a PRDM9-defined or a default hotspot.

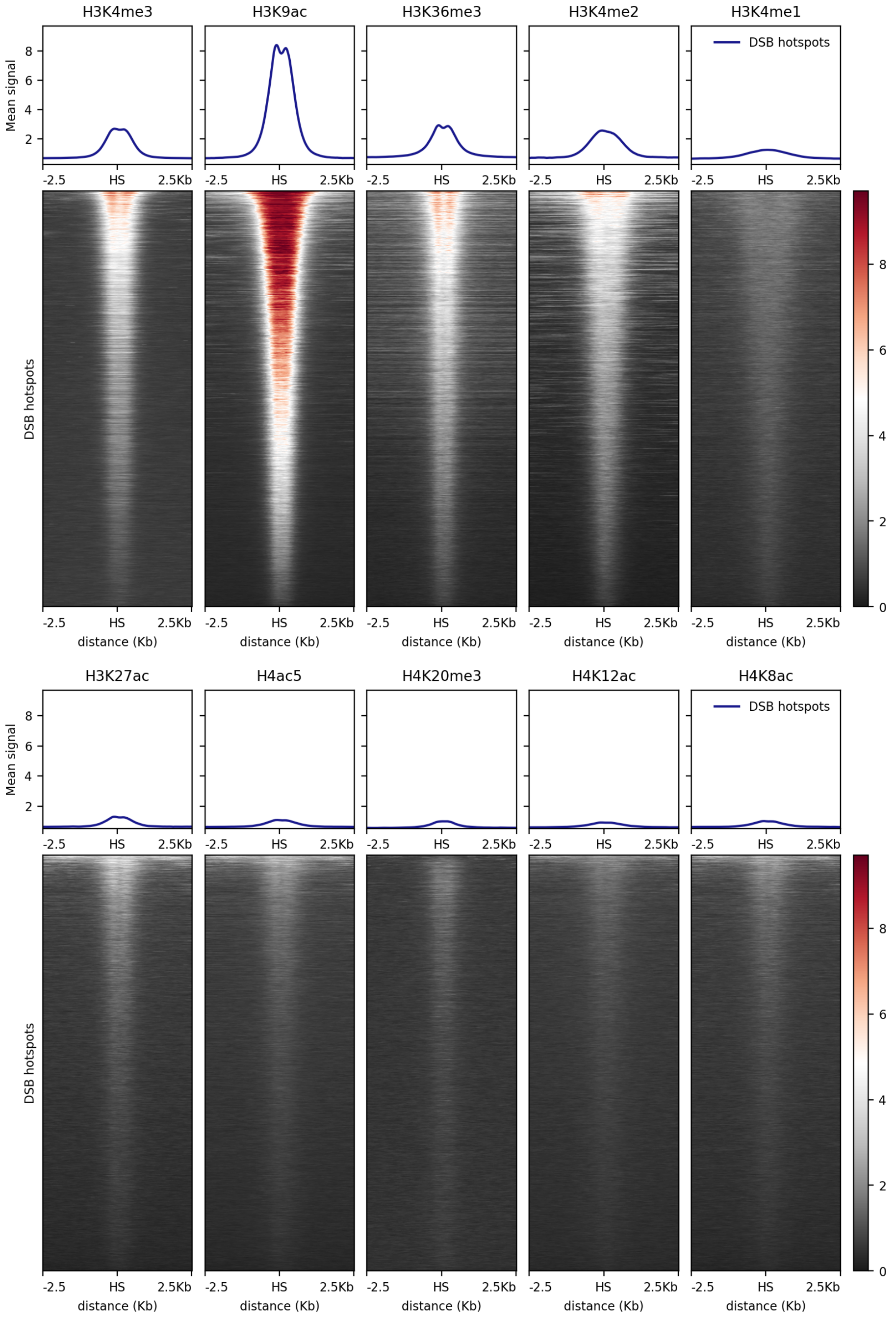
H3K4me3
H3K9ac
H3K36me3
H3K4me2
H3K4me1
DSB hotspots
Mean signal
DSB hotspots
distance (Kb)
-2.5
HS
2.5Kb
H3K27ac
H4ac5
H4K20me3
H4K12ac
H4K8ac

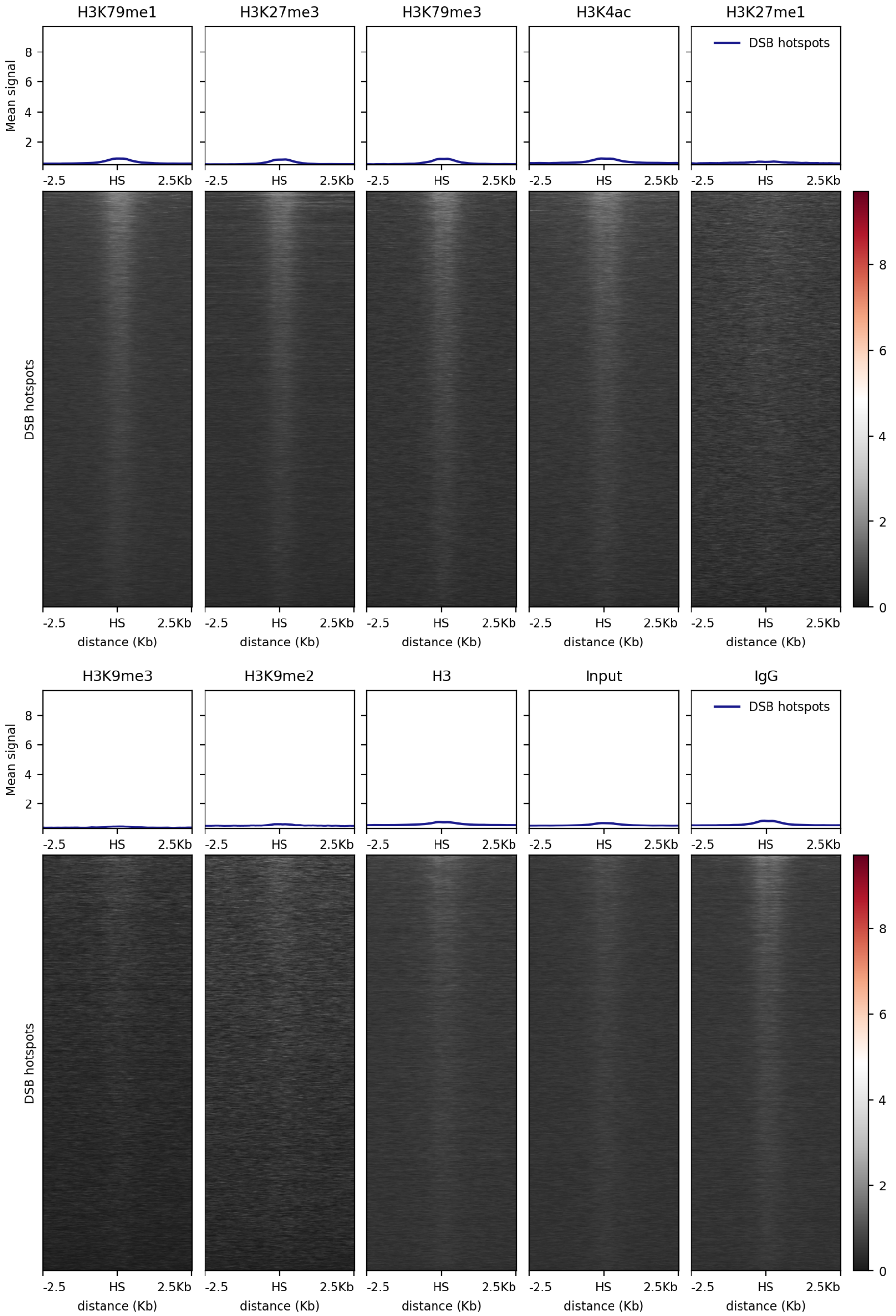

Supplementary Fig. 8 Heatmaps of histone modification ChIP-Seq at DSB hotspots. Coverage data were averaged in 150 bp windows and converted to reads per Kbp per million (RPKM) to facilitate cross-comparison. Heatmaps were generated using deeptools[3]. It is important to note that some enrichment at DSB hotspots is seen when using a non-specific antibody (IgG; last panel).

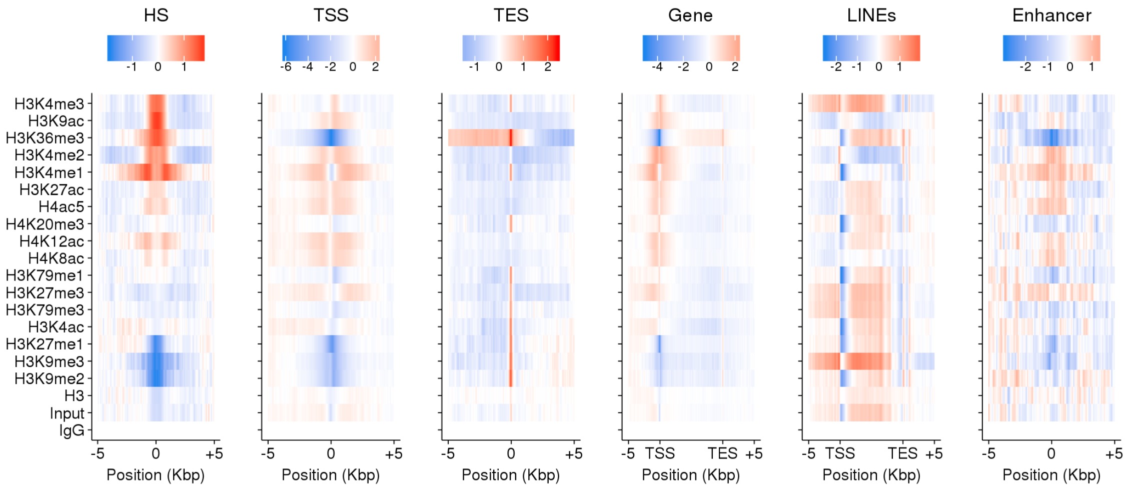

Supplementary Fig. 9 Enrichment of histone modifications at functional genomic elements. Histone modification coverage was plotted around functional sites in the genome. Enrichment is shown as mean signal - mean signal in IgG ChIP-Seq (red=high; blue=low). L1 LINEs were obtained from the repeatmasker database. Enhancers were obtained from the UCSC table browser RefSeq functional elements table.

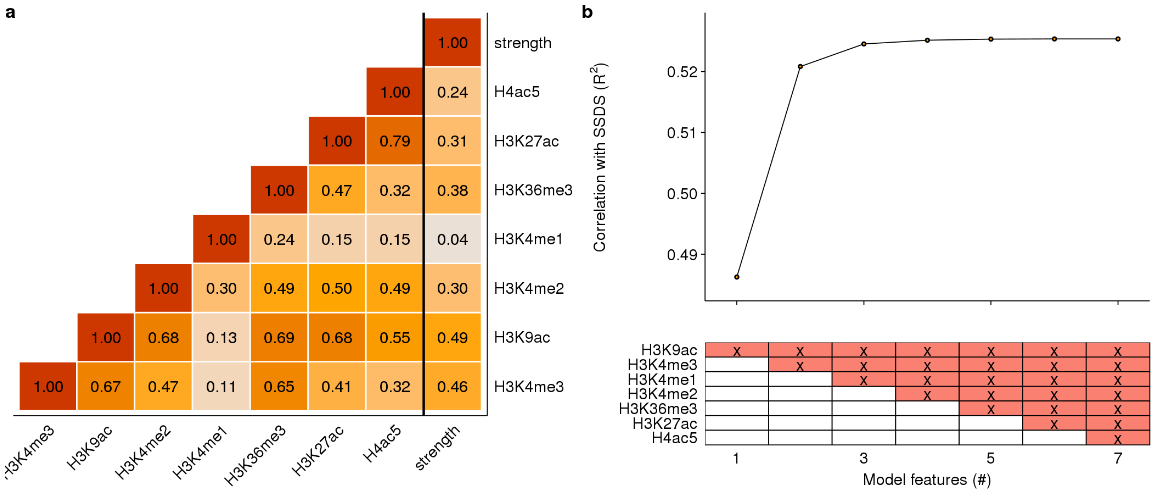

Supplementary Fig. 10 Correlations between histone modifications and DSB hotspot strength. a Pearson correlation coefficients of log-transformed read counts and hotspot strength (strength). Only histone marks enriched at DSB hotspots were considered. Hotspots with above-zero coverage for all histone marks and hotspot strength were used (N = 11,253). **b** Multiple linear regression with histone marks only marginally improves correlation with DSB hotspots. The lower panel depicts the histone marks used (red; x) for models with each number of features. The order of addition was determined using all-subsets regression with the leaps package in R. Small increases in R^2 may simply arise because additional samples reduce stochastic noise in strength estimates.

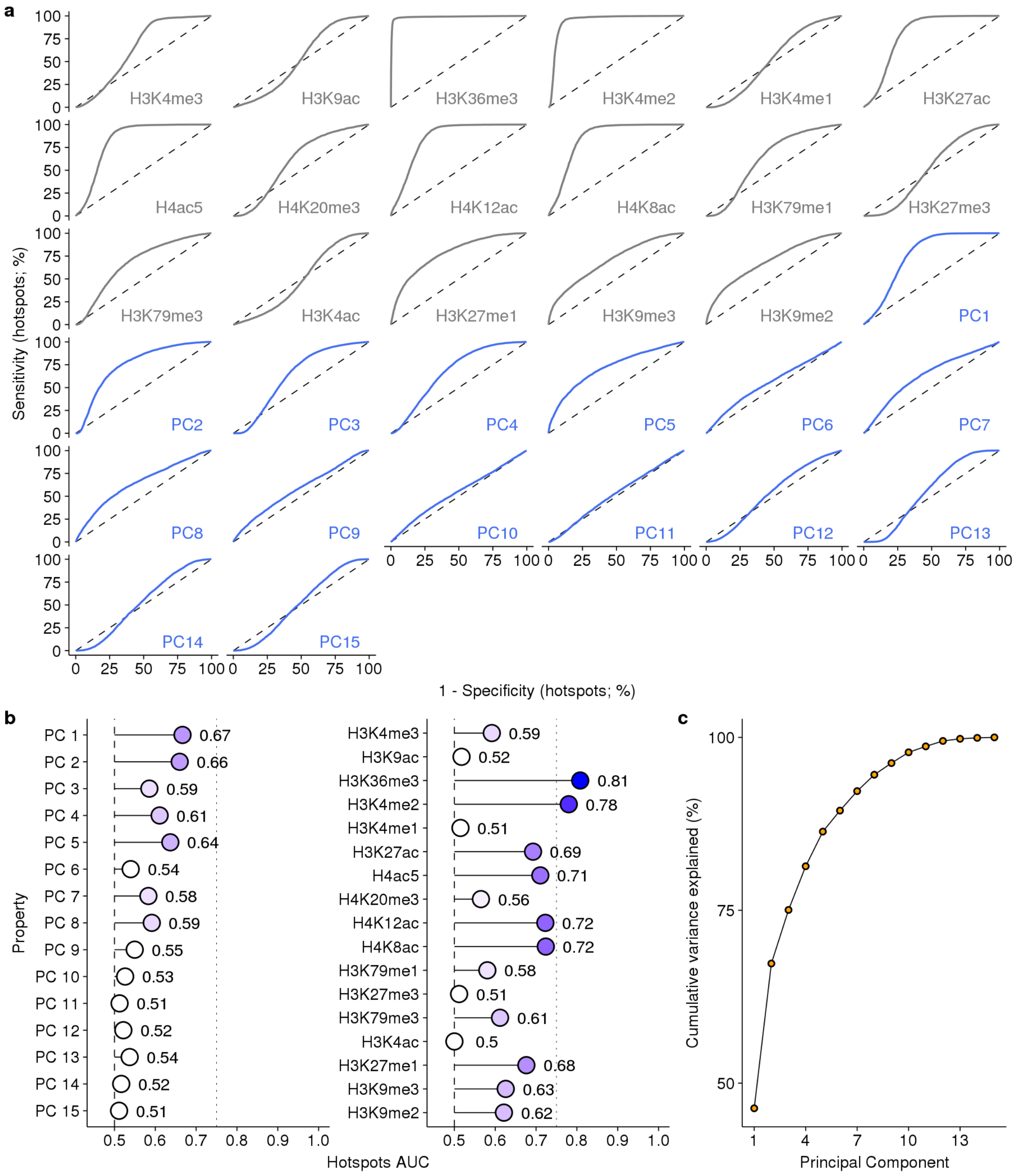

Supplementary Fig. 11: Histone marks distinguish hotspots from other H3K4me3 sites.

a ROC curves for individual histone marks and principal components (PCs) for discriminating hotspots from other H3K4me3 sites in the genome. Dashed line indicates no predictive ability. **b** The area under the ROC curves (AUC). **c** Most of the variance in histone mark signals is captured by few PCs.

Supplementary Table

Supplementary Table 1. Summary of ChIP conditions.

Sample	Replicate	Source	Shearing method	Starting chromatin DNA (ng)	ChIP volume (ul)	Antibody			
						Antigen	Brand	Catalog no.	Amount used (ug)
H3K4me3_Lep	1	Leptotene nuclei	sonication	2,398	1,000	H3K4me3	EpiCypher	13-0028	5
H3K4me3_Zyg	1	Zygotene nuclei	sonication	2,688	1,000	H3K4me3	EpiCypher	13-0028	5
H3K4me3_ePac	1	Early pachytene nuclei	sonication	1,793	1,000	H3K4me3	EpiCypher	13-0028	5
H3K4me3_lPac	1	Late pachytene nuclei	sonication	4,936	1,000	H3K4me3	EpiCypher	13-0028	5
H3K4me3_Dip	1	Diplotene nuclei	sonication	6,502	1,000	H3K4me3	EpiCypher	13-0028	5
H3K4me3_Lep	2	Leptotene nuclei	sonication	805	1,000	H3K4me3	EpiCypher	13-0028	1
H3K4me3_Zyg	2	Zygotene nuclei	sonication	755	1,000	H3K4me3	EpiCypher	13-0028	1
H3K4me3_ePac	2	Early pachytene nuclei	sonication	4,585	1,000	H3K4me3	EpiCypher	13-0028	1
H3K4me3_lPac	2	Late pachytene nuclei	sonication	915	1,000	H3K4me3	EpiCypher	13-0028	1
H3K4me3_Dip	2	Diplotene nuclei	sonication	1,115	1,000	H3K4me3	EpiCypher	13-0028	1
H3K4me3_Lep	2	Leptotene nuclei	sonication	805	1,000	H3K4me3	Abcam	ab8580	1
H3K4me3_Zyg	2	Zygotene nuclei	sonication	755	1,000	H3K4me3	Abcam	ab8580	1
H3K4me3_ePac	2	Early pachytene nuclei	sonication	960	1,000	H3K4me3	Abcam	ab8580	1
H3K4me3_lPac	2	Late pachytene nuclei	sonication	915	1,000	H3K4me3	Abcam	ab8580	1
H3K4me3_Dip	2	Diplotene nuclei	sonication	1,115	1,000	H3K4me3	Abcam	ab8580	1
Lep_input	1	Leptotene nuclei	sonication	48	NA	NA	NA	NA	NA
Zyg_input	1	Zygotene nuclei	sonication	54	NA	NA	NA	NA	NA
ePac_input	1	Early pachytene nuclei	sonication	36	NA	NA	NA	NA	NA
lPac_input	1	Late pachytene nuclei	sonication	99	NA	NA	NA	NA	NA
Dip_input	1	Diplotene nuclei	sonication	130	NA	NA	NA	NA	NA
Lep_input	2	Leptotene nuclei	sonication	16	NA	NA	NA	NA	NA
Zyg_input	2	Zygotene nuclei	sonication	15	NA	NA	NA	NA	NA
ePac_input	2	Early pachytene nuclei	sonication	92	NA	NA	NA	NA	NA
lPac_input	2	Late pachytene nuclei	sonication	18	NA	NA	NA	NA	NA
Dip_input	2	Diplotene nuclei	sonication	22	NA	NA	NA	NA	NA
H3K9ac_Lep	1	Leptotene nuclei	sonication	2,398	1,000	H3K9ac	Active motif	39918	1.5
H3K9ac_Zyg	1	Zygotene nuclei	sonication	2,688	1,000	H3K9ac	Active motif	39918	3
H3K9ac_ePac	1	Early pachytene nuclei	sonication	1,793	1,000	H3K9ac	Active motif	39918	3
H3K9ac_lPac	1	Late pachytene nuclei	sonication	4,936	1,000	H3K9ac	Active motif	39918	1.5
H3K9ac_Dip	1	Diplotene nuclei	sonication	6,502	1,000	H3K9ac	Active motif	39918	1.5
H3K9ac_Lep	2	Leptotene nuclei	sonication	601	800	H3K9ac	Active motif	39918	1
H3K9ac_Zyg	2	Zygotene nuclei	sonication	6,026	1,000	H3K9ac	Active motif	39918	1
H3K9ac_ePac	2	Early pachytene nuclei	sonication	4,585	1,000	H3K9ac	Active motif	39918	1.5
H3K9ac_lPac	2	Late pachytene nuclei	sonication	2,185	1,000	H3K9ac	Active motif	39918	1
H3K9ac_Dip	2	Diplotene nuclei	sonication	1,898	1,000	H3K9ac	Active motif	39918	1
No-antibody control	1	Leptotene nuclei	sonication	1,348	800	NA	NA	NA	NA
H3K4me3	1	Early (SCP3+H1t-) nuclei	MNase digestion	193	500	H3K4me3	Abcam	ab8580	1
H3K9ac	1	Early (SCP3+H1t-) nuclei	MNase digestion	193	500	H3K9ac	Active motif	39918	1
H3K4me2	1	Early (SCP3+H1t-) nuclei	MNase digestion	193	500	H3K4me2	Active motif	39914	1
H3K36me3	1	Early (SCP3+H1t-) nuclei	MNase digestion	193	500	H3K36me3	Active motif	61102	1
H3K4me1	1	Early (SCP3+H1t-) nuclei	MNase digestion	172	500	H3K4me1	Abcam	ab8895	1
H3K27ac	1	Early (SCP3+H1t-) nuclei	MNase digestion	193	500	H3K27ac	Abcam	ab177178	5
H4ac5	1	Early (SCP3+H1t-) nuclei	MNase digestion	193	500	H4ac5	Millipore	06-946	5ul
H4K8ac	1	Early (SCP3+H1t-) nuclei	MNase digestion	193	500	H4K8ac	Abcam	ab15823	5
H4K12ac	1	Early (SCP3+H1t-) nuclei	MNase digestion	193	500	H4K12ac	Active motif	39928	5
H4K20me3	1	Early (SCP3+H1t-) nuclei	MNase digestion	172	500	H4K20me3	Millipore	07-463	1
H3K4ac	1	Early (SCP3+H1t-) nuclei	MNase digestion	172	500	H3K4ac	Abcam	ab176799	1
H3K79me1	1	Early (SCP3+H1t-) nuclei	MNase digestion	172	500	H3K79me1	Abcam	ab2886	1
H3K79me3	1	Early (SCP3+H1t-) nuclei	MNase digestion	172	500	H3K79me3	Abcam	ab2621	1
H3K27me3	1	Early (SCP3+H1t-) nuclei	MNase digestion	172	500	H3K27me3	Millipore	07-449	1
H3K9me2	1	Early (SCP3+H1t-) nuclei	MNase digestion	172	500	H3K9me2	Abcam	ab1220	1
H3K9me3	1	Early (SCP3+H1t-) nuclei	MNase digestion	172	500	H3K9me3	Active motif	39766	1
H3K27me1	1	Early (SCP3+H1t-) nuclei	MNase digestion	172	500	H3K27me1	Millipore	07-448	1
H3	1	Early (SCP3+H1t-) nuclei	MNase digestion	193	500	H3	Abcam	ab1791	1
IgG	1	Early (SCP3+H1t-) nuclei	MNase digestion	193	500	IgG	Millipore	12-370	1
Input	1	Early (SCP3+H1t-) nuclei	MNase digestion	12	NA	NA	NA	NA	NA

Supplementary References

1. Shah, R. N. *et al.* Examining the Roles of H3K4 Methylation States with Systematically Characterized Antibodies. *Mol. Cell* **72**, 162–177.e7 (2018).
2. Brick, K., Smagulova, F., Khil, P., Camerini-Otero, R. D. & Petukhova, G. V. Genetic recombination is directed away from functional genomic elements in mice. *Nature* **485**, 642–645 (2012).
3. Ramírez, F., Dündar, F., Diehl, S., Grüning, B. A. & Manke, T. deepTools: a flexible platform for exploring deep-sequencing data. *Nucleic Acids Res.* **42**, W187–91 (2014).
