# Supplementary material for: Cell-type-specific genomics reveals histone modification dynamics in mammalian meiosis
Source: Nat Commun. 2019 Aug 23;10:3821. doi: 10.1038/s41467-019-11820-7 (PMC6707301; doi:10.1038/s41467-019-11820-7)
Supplement: Supplementary file 3 — Reporting Summary [file 41467_2019_11820_MOESM3_ESM.pdf]

nature research

Corresponding author(s): R. Daniel Camerini-Otero

Reporting Summary

Nature Research wishes to improve the reproducibility of the work that we publish. This form provides structure for consistency and transparency in reporting. For further information on Nature Research policies, see Authors & Referees and the Editorial Policy Checklist.

Statistical parameters

When statistical analyses are reported, confirm that the following items are present in the relevant location (e.g. figure legend, table legend, main text, or Methods section).

n/a	Confirmed	
☐	☒	The exact sample size (*n*) for each experimental group/condition, given as a discrete number and unit of measurement
☐	☒	An indication of whether measurements were taken from distinct samples or whether the same sample was measured repeatedly
☐	☒	The statistical test(s) used AND whether they are one- or two-sided<br>*Only common tests should be described solely by name; describe more complex techniques in the Methods section.*
☒	☐	A description of all covariates tested
☐	☒	A description of any assumptions or corrections, such as tests of normality and adjustment for multiple comparisons
☐	☒	A full description of the statistics including central tendency (e.g. means) or other basic estimates (e.g. regression coefficient) AND variation (e.g. standard deviation) or associated estimates of uncertainty (e.g. confidence intervals)
☒	☐	For null hypothesis testing, the test statistic (e.g. *F*, *t*, *r*) with confidence intervals, effect sizes, degrees of freedom and *P* value noted<br>*Give P values as exact values whenever suitable.*
☒	☐	For Bayesian analysis, information on the choice of priors and Markov chain Monte Carlo settings
☒	☐	For hierarchical and complex designs, identification of the appropriate level for tests and full reporting of outcomes
☒	☐	Estimates of effect sizes (e.g. Cohen's *d*, Pearson's *r*), indicating how they were calculated
☐	☒	Clearly defined error bars<br>*State explicitly what error bars represent (e.g. SD, SE, CI)*

Our web collection on statistics for biologists may be useful.

Software and code

Policy information about availability of computer code

Data collection	We used Illumina pipelines for image processing and base calling to collect our data.
Data analysis	We used NCIS to do background correction for some ChIP-Seq samples. We called peaks using MACS2 (version 2.1.2) with default parameters except (-q 0.1 --broad). We used bedtools (version v2.27.1) to determine if peaks overlap with DSB hotspots and/or gene promoters. We used R factoextra package, R leaps package, and R prcomp command to perform downstream analyses. All custom codes for data analysis are available at https://doi.org/10.5281/zenodo.2651204.

For manuscripts utilizing custom algorithms or software that are central to the research but not yet described in published literature, software must be made available to editors/reviewers upon request. We strongly encourage code deposition in a community repository (e.g. GitHub). See the Nature Research guidelines for submitting code & software for further information.

Data

Policy information about availability of data

All manuscripts must include a data availability statement. This statement should provide the following information, where applicable:

- Accession codes, unique identifiers, or web links for publicly available datasets
- A list of figures that have associated raw data
- A description of any restrictions on data availability

The sequencing data reported in this manuscript are archived at the Gene Expression Omnibus (www.ncbi.nlm.nih.gov/geo) as accession no. GSE121760. The source data for all figures are provided as a Source Data file.

Field-specific reporting

Please select the best fit for your research. If you are not sure, read the appropriate sections before making your selection.

☒ Life sciences ☐ Behavioural & social sciences ☐ Ecological, evolutionary & environmental sciences

For a reference copy of the document with all sections, see nature.com/authors/policies/ReportingSummary-flat.pdf

Life sciences study design

All studies must disclose on these points even when the disclosure is negative.

Sample size	Sample-size calculation was not performed for the sorting experiments. The amounts of chromatin DNA used for each ChIP-Seq experiment have been reported in Methods and Supplementary Table 1.
Data exclusions	The details and the rationale of data exclusion for specific bioinformatic analyses have been described in Methods.
Replication	Two or three replicates were performed on sorting specific nuclei populations. All attempts of replicates were successful with similar purity (reported in Table 1). Replicates were performed for stage-specific H3K4me3/H3K9ac-ChIP-Seq.
Randomization	Randomization was not performed for isolating nuclei. Randomizations for some bioinformatic studies were described in Methods.
Blinding	Blinding was not performed during data collection.

Reporting for specific materials, systems and methods

Materials & experimental systems

n/a	Involved in the study	
☒	☐	Unique biological materials
☐	☒	Antibodies
☒	☐	Eukaryotic cell lines
☒	☐	Palaeontology
☐	☒	Animals and other organisms
☒	☐	Human research participants

Methods

n/a	Involved in the study	
☐	☒	ChIP-seq
☐	☒	Flow cytometry
☒	☐	MRI-based neuroimaging

Antibodies

Antibodies used

1. Anti-SCP3; supplier name: Santa Cruz Biotechnology; catalog number: sc-74569; clone name: D-1; lot number: D0717
2. Anti-Stra8; supplier name: abcam; catalog number: ab49602; lot number: GR275803
3. Anti-H1t; supplier names: a gift from Mary Ann Handel and a custom-made antibody from Neo-Bioscience
4. Anti-SCP1; supplier name: Novus Biologicals; catalog number: NB300-299B; lot number: D1-070617-B
5. Anti-H3K4me3; supplier name: abcam; catalog number: ab8580; lot number: GR565368
6. Anti-H3K4me3; supplier name: EpiCypher; catalog number: 13-0028; lot number: 18303001
7. Anti-H3K9ac; supplier name: Active Motif; catalog number: 39918; lot number: 23913004
8. Anti-H3K4me2; supplier name: Active Motif; catalog number: 39914; lot number: 04812002
9. Anti-H3K36me3; supplier name: Active Motif; catalog number: 61102; lot number: 32412503
10. Anti-H4ac5; supplier name: Millipore; catalog number: 06-946; lot number: 2631278
11. Anti-H3K4me1; supplier name: abcam; catalog number: ab889; lot number: GR283603

12. Anti-H2K27ac; supplier name: abcam; catalog number: ab177178; clone name: EP16602; lot number: GR262881
13. Anti-H4K8ac; supplier namer: abcam; catalog number: ab15823; lot number: GR287803
14. Anti-H4K12ac; supplier name: Active Motif; catalog number: 39928; lot number: 27710001
15. Anti-H3K27me3; supplier name: Millipore; catalog number: 07-449; lot number: 2736613
16. Anti-H4K20me3; supplier name: Millipore; catalog number: 07-463; lot number: 2727201
17. Anti-H3K79me1; supplier name: abcam; catalog number: ab2886; lot number: GR274715
18. Anti-H3K4ac; supplier name: abcam; catalog number: ab176799; clone name: EPR16596; lot number: GR226364
19. Anti-H3K79me3; supplier name: abcam; catalog number: ab2621; lot number: GR277773
20. Anti-H3K9me2; supplier name: abcam; catalog number: ab1220; lot number: GR212253
21. Anti-H3K9me3; supplier name: Active Motif; catalog number: 39766; lot number: 16513004
22. Anti-H3K27me1; supplier name: Millipore; catalog number: 07-448; lot number: 2702115
23. Anti-H3; supplier name: abcam; catalog number: ab1791; lot number: GR300976
24. Normal rabbit IgG; supplier name: Millipore; catalog number: 12-370; lot number: 2603252

Validation

1. The anti-SCP3 antibody is a mouse monoclonal IgG1. We used this antibody as a marker for nuclei sorting. This antibody has been cited in >64 citations according to the manufacturer's website.
2. The anti-Stra8 is a polyclonal rabbit IgG. We used this antibody as a marker for nuclei sorting. This antibody has been referenced in 37 publications according to the manufacturer's website.
3. We used the H1t antibodies as a marker for nuclei sorting. Handel's anti-H1t antibody was from guinea pig serum and was published in Inselman, et. al. (2003) Temporal expression of cell cycle-related proteins during spermatogenesis: establishing a timeline for onset of the meiotic divisions. Cytogenet Genome Res 103:277-284. The custom-made H1t antibody is a rabbit polyclonal IgG. It was validated using immunofluorescence staining in spermatocyte spreads compared in parallel with Handel's H1t antibody.
4. The anti-SCP1 antibody is a biotin-conjugated polyclonal rabbit IgG. We used this antibody as a marker for nuclei sorting.
5. The anti-H3K4me3 antibody is a polyclonal rabbit IgG. We used this antibody for ChIP-Seq. This antibody has been referenced in 1118 publications according to the manufacturer's website.
6. The anti-H3K4me3 antibody is a monoclonal rabbit IgG. We used this antibody for ChIP-Seq. This antibody is highly specific and has been "SNAP-ChIP" certified according to the manufacturer's website.
7. The anti-H3K9ac antibody is a polyclonal rabbit IgG. We used this antibody for ChIP-Seq. This antibody has been referenced in 18 publications according to the manufacturer's website.
8. The anti-H3K4me2 antibody is a polyclonal rabbit IgG. We used this antibody for ChIP-Seq. This antibody has been referenced in one publication according to the manufacturer's website.
9. The anti-H3K36me3 antibody is a monoclonal rabbit IgG. We used this antibody for ChIP-Seq. This antibody has been referenced in 17 publications according to the manufacturer's website.
10. The anti-H4ac5 antibody is rabbit polyclonal antiserum IgG. We used this antibody for ChIP-Seq. This antibody has been referenced in 42 publications according to the manufacturer's website.
11. The anti-H3K4me1 antibody is a polyclonal rabbit IgG. We used this antibody for ChIP-Seq. This antibody has been referenced in 540 publications according to the manufacturer's website.
12. The anti-H3K7ac antibody is a polyclonal rabbit IgG. We used this antibody for ChIP-Seq. This antibody has been referenced in 4 publications according to the manufacturer's website.
13. The anti-H4K8ac antibody is a polyclonal rabbit IgG. We used this antibody for ChIP-Seq. This antibody has been referenced in 39 publications according to the manufacturer's website.
14. The anti-H4K12ac antibody is a polyclonal rabbit IgG. We used this antibody for ChIP-Seq. This antibody has been referenced in 2 publications according to the manufacturer's website.
15. The anti-H3K27me3 antibody is a polyclonal rabbit IgG. We used this antibody for ChIP-Seq. This antibody has been referenced in >50 publications according to the manufacturer's website.
16. The anti-H4K20me3 antibody is a polyclonal rabbit IgG. We used this antibody for ChIP-Seq. This antibody has been referenced in >50 publications according to the manufacturer's website.
17. The anti-H3K79me1 antibody is a polyclonal rabbit IgG. We used this antibody for ChIP-Seq. This antibody has been referenced in 41 publications according to the manufacturer's website.
18. The anti-H3K4ac antibody is a monoclonal rabbit IgG. We used this antibody for ChIP-Seq.
19. The anti-H3K79me3 antibody is a polyclonal rabbit IgG. We used this antibody for ChIP-Seq. This antibody has been referenced in 102 publications according to the manufacturer's website.
20. The anti-H3K9me2 antibody is a polyclonal rabbit IgG. We used this antibody for ChIP-Seq. This antibody has been referenced in 475 publications according to the manufacturer's website.
21. The anti-H3K9me3 antibody is a polyclonal rabbit IgG. We used this antibody for ChIP-Seq. This antibody has been referenced in 13 publications according to the manufacturer's website.
22. The anti-H3K27me1 antibody is a polyclonal rabbit IgG. We used this antibody for ChIP-Seq. This antibody has been referenced in >50 publications according to the manufacturer's website.
23. The anti-H3 antibody is a polyclonal rabbit IgG. We used this antibody for ChIP-Seq. This antibody has been referenced in 2233 publications according to the manufacturer's website.
24. We used the normal rabbit IgG for ChIP-Seq. This antibody has been referenced in >50 publications according to the manufacturer's website.

Animals and other organisms

Policy information about studies involving animals; ARRIVE guidelines recommended for reporting animal research

Laboratory animals

C57BL/6J (B6) mice were either obtained from The Jackson Laboratory (Stock no. 000664) or bred in-house. All experiments were done on adult mice (>= 8 weeks of age).

Wild animals

This study did not involve wild animals.

Field-collected samples

This study did not involve samples collected from the field.

ChIP-seq

Data deposition

☒ Confirm that both raw and final processed data have been deposited in a public database such as GEO.

☒ Confirm that you have deposited or provided access to graph files (e.g. BED files) for the called peaks.

Data access links
May remain private before publication.

The sequencing data are available for review at the GEO https://www.ncbi.nlm.nih.gov/geo/query/acc.cgi?acc=GSE121760

Files in database submission

LE1_H3K4me3_peaks.bed
LE2_H3K4me3_peaks.bed
ZY1_H3K4me3_peaks.bed
ZY2_H3K4me3_peaks.bed
EP1_H3K4me3_peaks.bed
EP2_H3K4me3_peaks.bed
LP1_H3K4me3_peaks.bed
LP2_H3K4me3_peaks.bed
DI1_H3K4me3_peaks.bed
DI2_H3K4me3_peaks.bed
LE1_H3K4me3.mm10.TPM.bigwig
LE2_H3K4me3.mm10.TPM.bigwig
LE_rep1_H3K9ac.mm10.TPM.bigwig
LE_rep1_input.mm10.RPKM.bigwig
ZY1_H3K4me3.mm10.TPM.bigwig
ZY2_H3K4me3.mm10.TPM.bigwig
ZY_rep1_H3K9ac.mm10.TPM.bigwig
ZY_rep1_input.mm10.RPKM.bigwig
EP1_H3K4me3.mm10.TPM.bigwig
EP2_H3K4me3.mm10.TPM.bigwig
EP_rep1_H3K9ac.mm10.TPM.bigwig
EP_rep1_input.mm10.RPKM.bigwig
LP1_H3K4me3.mm10.TPM.bigwig
LP2_H3K4me3.mm10.TPM.bigwig
LP_rep1_H3K9ac.mm10.TPM.bigwig
LP_rep1_input.mm10.RPKM.bigwig
DI1_H3K4me3.mm10.TPM.bigwig
DI2_H3K4me3.mm10.TPM.bigwig
DI_rep1_H3K9ac.mm10.TPM.bigwig
DI_rep1_input.mm10.RPKM.bigwig
H3K27ac_SCP3pos_H1Tneg.TPM.bigwig
H3K27me1_SCP3pos_H1Tneg.TPM.bigwig
H3K27me3_SCP3pos_H1Tneg.TPM.bigwig
H3K36me3_SCP3pos_H1Tneg.TPM.bigwig
H3K4ac_SCP3pos_H1Tneg.TPM.bigwig
H3K4me1_SCP3pos_H1Tneg.TPM.bigwig
H3K4me2_SCP3pos_H1Tneg.TPM.bigwig
H3K4me3_SCP3pos_H1Tneg.TPM.bigwig
H3K79me1_SCP3pos_H1Tneg.TPM.bigwig
H3K79me3_SCP3pos_H1Tneg.TPM.bigwig
H3K9ac_SCP3pos_H1Tneg.TPM.bigwig
H3K9me2_SCP3pos_H1Tneg.TPM.bigwig
H3K9me3_SCP3pos_H1Tneg.TPM.bigwig
H3_SCP3pos_H1Tneg.TPM.bigwig
H4K12ac_SCP3pos_H1Tneg.TPM.bigwig
H4K20me3_SCP3pos_H1Tneg.TPM.bigwig
H4K8ac_SCP3pos_H1Tneg.TPM.bigwig
H4ac5_SCP3pos_H1Tneg.TPM.bigwig
IgG_SCP3pos_H1Tneg.TPM.bigwig
input_SCP3pos_H1Tneg.TPM.bigwig
LE1_H3K4me3.mm10.fastq.gz
LE2_H3K4me3.mm10.fastq.gz
LE_rep1_H3K9ac.mm10.fastq.gz
LE_rep1_input.mm10.fastq.gz
ZY1_H3K4me3.mm10.fastq.gz
ZY2_H3K4me3.mm10.fastq.gz
ZY_rep1_H3K9ac.mm10.fastq.gz
ZY_rep1_input.mm10.fastq.gz
EP1_H3K4me3.mm10.fastq.gz
EP2_H3K4me3.mm10.fastq.gz
EP_rep1_H3K9ac.mm10.fastq.gz

EP_rep1_input.mm10.fastq.gz
LP1_H3K4me3.mm10.fastq.gz
LP2_H3K4me3.mm10.fastq.gz
LP_rep1_H3K9ac.mm10.fastq.gz
LP_rep1_input.mm10.fastq.gz
DI1_H3K4me3.mm10.fastq.gz
DI2_H3K4me3.mm10.fastq.gz
DI_rep1_H3K9ac.mm10.fastq.gz
DI_rep1_input.mm10.fastq.gz
H3K27ac_SCP3pos_H1Tneg.fastq.gz
H3K27me1_SCP3pos_H1Tneg.fastq.gz
H3K27me3_SCP3pos_H1Tneg.fastq.gz
H3K36me3_SCP3pos_H1Tneg.fastq.gz
H3K4ac_SCP3pos_H1Tneg.fastq.gz
H3K4me1_SCP3pos_H1Tneg.fastq.gz
H3K4me2_SCP3pos_H1Tneg.fastq.gz
H3K4me3_SCP3pos_H1Tneg.fastq.gz
H3K79me1_SCP3pos_H1Tneg.fastq.gz
H3K79me3_SCP3pos_H1Tneg.fastq.gz
H3K9ac_SCP3pos_H1Tneg.fastq.gz
H3K9me2_SCP3pos_H1Tneg.fastq.gz
H3K9me3_SCP3pos_H1Tneg.fastq.gz
H3_SCP3pos_H1Tneg.fastq.gz
H4K12ac_SCP3pos_H1Tneg.fastq.gz
H4K20me3_SCP3pos_H1Tneg.fastq.gz
H4K8ac_SCP3pos_H1Tneg.fastq.gz
H4ac5_SCP3pos_H1Tneg.fastq.gz
IgG_SCP3pos_H1Tneg.fastq.gz
input_SCP3pos_H1Tneg.fastq.gz

Genome browser session (e.g. UCSC)

We have deposited bigwig files at the GEO.

Methodology

Replicates

Three replicates of each stage-specific H3K4me3-ChIP-Seq were performed and details for each experiment were provided in Supplementary Table 1. Two replicates were performed for stage-specific H3K9ac-ChIP-Seq.

Sequencing depth

All samples were either single-end or pair-end sequenced. The read length of single-end and pair-end sequencing was 51-bp and 151-bp, respectively. The uniquely mapped reads of these samples range from 6 to 167 millions.

Antibodies

Described above.

Peak calling parameters

Peaks for H3K4me3 ChIP-Seq were called using MACS2 (version 2.1.2) with default parameters except (-q 0.1 --broad) and with a stage-matched input DNA sample as a control. Peaks overlapping DSB hotspots or gene promoters were ascertained using bedtools (version v2.27.1) after removing blacklisted regions.

Data quality

The details of data quality including number of called peaks and fold-enrichment for the stage-specific H3K4me3-ChIP-Seq have been described in Table 1 in the manuscript. The data quality of ChIP-Seq for other histone modifications has been described in the main text and specific details have been reported in Supplementary Fig 9.

Software

We aligned sequencing tags to the mouse mm10 reference genome using BWA mem 0.7.12. We used NCIS to do background correction. We called peaks using MACS2 (version 2.1.2). We used bedtools (version v2.27.1) to determine if peaks overlap with DSB hotspots and/or gene promoters. We used R factoextra package, R leaps package, and R prcomp command to perform downstream analyses. All custom codes for data analysis are available at https://doi.org/10.5281/zenodo.2651204.

Flow Cytometry

Plots

Confirm that:

☒ The axis labels state the marker and fluorochrome used (e.g. CD4-FITC).

☒ The axis scales are clearly visible. Include numbers along axes only for bottom left plot of group (a 'group' is an analysis of identical markers).

☒ All plots are contour plots with outliers or pseudocolor plots.

☒ A numerical value for number of cells or percentage (with statistics) is provided.

Methodology

Sample preparation

Described in Methods.

Instrument	BD FACSAria II or BD FACSAria Fusion
Software	FlowJo v10 was used for flow cytometric data analysis.
Cell population abundance	The abundance and purity of the relevant nuclei populations were described in details in Results and Methods.
Gating strategy	We gated singlets using both SSC and FSC gates. A distinct population of meiotic 4C nuclei population was selected using DAPI and SCP3 antibody (shown in Fig. 2b). 1C nuclei are SCP3 negative and were used as a negative control for gate setting. Leptonema are the only STRA8-positive 4C meiocytes. We isolated leptotene nuclei from other meiotic 4C nuclei using STRA8 antibody. 1C nuclei are STRA8 negative and were used as a negative control for gate setting. Boundaries of selecting the other four meiotic sub-populations were shown in Fig. 2b. The x-axis represents the H1t signal (FITC fluorescence), whereas the y-axis represents the SCP1 signal (AF647 fluorescence).

☒ Tick this box to confirm that a figure exemplifying the gating strategy is provided in the Supplementary Information.
